# Alkoxy-Bridged Dicopper(II) Cores Meet Tetracyanonickelate Linkers: Structural, Magnetic, and Theoretical Investigation of Cu/Ni Coordination Polymers

**DOI:** 10.1021/acs.jpcc.3c08112

**Published:** 2024-04-02

**Authors:** Inês F. M. Costa, Chris H. J. Franco, Dmytro S. Nesterov, Vânia André, Laura C. J. Pereira, Alexander M. Kirillov

**Affiliations:** † Centro de Química Estrutural, Institute of Molecular Sciences, Departamento de Engenharia Química, Instituto Superior Técnico, Universidade de Lisboa, Av. Rovisco Pais, 1049-001 Lisbon, Portugal; ‡ Centro de Ciências e Tecnologias Nucleares, Departmento de Engenharia Ciências Nucleares, Instituto Superior Técnico, Universidade de Lisboa, Estrada Nacional 10, 2695-066 Bobadela, Portugal

## Abstract

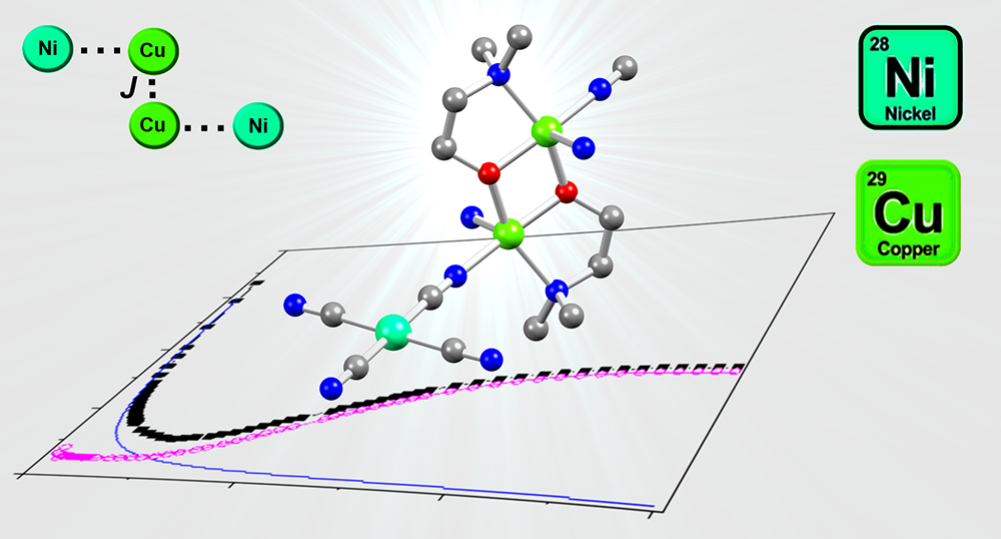

Two heterometallic Cu(II)/Ni(II) coordination polymers, [Cu_2_(Hbdea)_2_Ni(CN)_4_]_*n*_ (**1**) and [Cu_2_(dmea)_2_Ni(CN)_4_]_*n*_·*n*H_2_O (**2**), were successfully self-assembled in water by reacting Cu(II) nitrate with H_2_bdea (*N*-butyldiethanolamine) and Hdmea (*N*,*N*-dimethylethanolamine) in the presence of sodium hydroxide and [Ni(CN)_4_]^2–^. These new coordination polymers were investigated by single-crystal and powder X-ray diffraction and fully characterized by FT-IR spectroscopy, thermogravimetry, elemental analysis, variable-temperature magnetic susceptibility measurements, and theoretical DFT and CASSCF calculations. Despite differences in crystal systems, in both compounds, each dinuclear building block [Cu_2_(μ-aminopolyalcoholate)_2_]^2+^ is bridged by diamagnetic [Ni(CN)_4_]^2–^ linkers, resulting in 1D (**1**) or 2D (**2**) metal–organic architectures. Experimental magnetic studies show that both compounds display strong antiferromagnetic coupling (*J* = −602.1 cm^–1^ for **1** and −151 cm^–1^ for **2**) between Cu(II) ions within the dimers mediated by the μ-O-alkoxo bridges. These results are corroborated by the broken symmetry DFT studies, which also provide further insight into the electronic structures of copper dimeric units. By reporting a facile self-assembly synthetic protocol, this study can be a model to widen a still limited family of heterometallic Cu/Ni coordination polymer materials with different functional properties.

## 1 Introduction

Heterometallic coordination polymers (CPs) incorporating magnetically active building blocks have gained increased attention in recent years because of possible cooperative phenomena of the magnetic spins.^1−7^ The strongest magnetic coupling between the spins of the nearest-neighbor paramagnetic centers is typically mediated by short bridging ligands, including oxo, cyano, azido, or carboxylate moieties, which have extensively been studied due to their ability to transmit magnetic exchange of different nature and magnitude.^8−14^ Considering the dependence of the magnetic interactions on the type of metal ion and the superexchange path, the selection of transition metals and bridging groups emerges as a key feature in the construction of heterometallic coordination systems.^15,16^

Systemic research has been conducted on the coordination polymers driven by the [M(CN)_*x*_] (M = Fe, Co, Ni, Cu, or Pd; *x* = 4 or 6) linkers.^17^ In this context, cyanometalate anions including [Fe(CN)_6_]^3–^,^18−21^ [Co(CN)_6_]^3–^,^11,22−27^ [Ni(CN)_4_]^2–^,^27−37^ or [Pd(CN)_4_]^2–^^29,33,38^ are particularly interesting given their ability to connect to other metal centers through a different number of cyanide groups, producing magnetically attractive heterometallic systems. However, the use of square-planar building blocks such as tetracyanonickellate(II) anions in the design of heterometallic systems containing multidentate aminoalcohols as stabilizing ligands has been underexplored. Such Cu/Ni systems can benefit from different coordination geometries of Ni(II) and Cu(II) centers^39^ and the formation of Cu-aminoalcoholate cations with μ-O-alkoxo bridges as possible magnetic coupling mediators. A search of the Cambridge Structural Database (CSD version 5.45 updated, Nov 2023) returned only four crystal structures of compounds comprising copper ions, [Ni(CN)_4_]^2–^, and an aminoalcohol ligand with the following formulas: {[Cu(hedt)_2_][Ni(CN)_4_]},^40^ {[Cu_2_(aeea)_2_][Ni(CN)_4_]}_*n*_,^35^ {[Cu(dien)(mea)][Ni(CN)_4_]·2H_2_O},^41^ and {[Cu_2_(pa)_2_][Ni(CN)_4_]}_*n*_,^42^ of which only two are the coordination polymers with dicopper(II) aminoalcoholate units {hedt = *N*,*N*-bis(2-hydroxyethyl)ethylenediamine, aeea = 2-((2-aminoethyl)amino)ethan-1-ol, dien = *N*-(2-aminoethyl)-1,2-ethanediamine, mea = 2-aminoethanol, pa = propanolamine). Despite being potentially interesting in terms of magnetic properties, these were investigated for only one compound, {[Cu_2_(aeea)_2_][Ni(CN)_4_]}_*n*_, which revealed a very strong antiferromagnetic interaction between Cu(II) ions.

An appealing room temperature self-assembly approach to generate heterometallic CPs in aqueous medium and using simple chemicals along with a magnetic attractiveness of the resulting systems prompted us to explore the self-assembly reactions between copper(II) ions, multidentate aminoalcohols, and tetracyanonickelate linkers.^43−45^ Hence, in the present work, we describe the synthesis and characterization of two new cyanide-bridged Cu(II)/Ni(II) coordination polymers formulated as [Cu_2_(Hbdea)_2_Ni(CN)_4_]_*n*_ (**1**) and [Cu_2_(dmea)_2_Ni(CN)_4_]_*n*_·H_2_O (**2**). These products were assembled from Cu(II) nitrate, *N*-butyldiethanolamine (H_2_bdea), or *N*,*N*-dimethylethanolamine (Hdmea) as *N*,*O*-donor supporting ligands and K_2_[Ni(CN)_4_] as a linker source. The structural features, magnetic susceptibility, and broken symmetry DFT studies of the obtained materials are discussed herein in detail. By reporting a facile self-assembly synthetic protocol, this work can serve as a model to broaden a still limited family of heterometallic Cu/Ni coordination polymers with different functional properties.

## 2 Methods

### 2.1 Synthetic Procedure for **1** and **2**

The corresponding aminoalcohol (*N*-butyldiethanolamine, 1 mmol, 163 μL for **1** or *N,N*-dimethylethanolamine, 1 mmol, 100 μL for **2**) was added under stirring to an aqueous solution of Cu(NO_3_)_2_·2.5H_2_O (1 mmol, 233 mg, 10 mL) containing HNO_3_ (0.1 M, 10 mL). An aqueous solution of NaOH (6 mL, 6 mmol) was then added dropwise and under constant stirring to the reaction mixture, followed by the introduction of potassium tetracyanonickelate(II) (K_2_[Ni(CN)_4_], 0.2 mmol, 48.2 mg), previously dissolved in 3 mL of H_2_O. The resulting green solution was stirred for 45 min before being filtered off. The filtrate was kept in an open vial for gradual evaporation at room temperature. The crystallization process occurred within 2–4 weeks, leading to the formation of green single-crystals suitable for X-ray analysis (Figure S1, Supporting Information). These were then collected and air-dried to provide the as-synthesized coordination polymers (∼35% yield of **1** or **2** based on copper(II) nitrate). [Cu_2_(Hbdea)_2_Ni(CN)_4_]_*n*_ (CP code **1**). FTIR-ATR (cm^–1^): 2990 (m), 2940 (m), 2880 (m) ν(CH), 2178 (vs) and 2140 (vs) ν(CN), 1453 (w), 1223 (w), 1090 (vs), 1025 (vs), 983 (s), 898 (s), and 740 (w). Anal. Calcd for **1**+H_2_O: C, 38.35%; H, 5.79%; N, 13.42%. Found: C, 37.91%; H, 5.62%; N, 13.09%. [Cu_2_(dmea)_2_Ni(CN)_4_]_*n*_·*n*H_2_O (CP **2**). FTIR-ATR (cm^–1^): 3440 (m) ν(OH/H_2_O), 2168 (vs) and 2150 (s) ν(CN), 1636 (w) δ(H_2_O), 1465 (m), 1200 (m), 1084 (vs), 950 (vs), 907 (vs), and 781 (s). Anal. Calcd for **2**+H_2_O: C, 28.70%; H, 4.82%; N, 16.74%. Found: C, 28.58%; H, 4.34%, N, 16.38%. Both compounds **1** and **2** are hygroscopic when stored in air, and their elemental and thermal analyses may reveal the presence of additional water molecules.

### 2.2 Single-Crystal X-ray Diffraction

Data for **1** were collected on an XtaLAB Synergy diffractometer with a HyPix-Arc 150° detector using Cu Kα radiation (λ = 1.54184 Å; 3D mirrors). All data were integrated by CrysAlisPro 1.171.42.49,^46^ and an absorption correction was applied using the integrated multiscan absorption algorithm. For **2**, the diffraction data were obtained on a Bruker APEX-II diffractometer with CCD detector using Mo Kα radiation (λ = 0.71073 Å). APEX programs were used for data collection, while SAINT and SADABS were applied for absorption correction.^47^ The crystal structures were solved by Direct Methods (SHELXT)^48^ and refined by full-matrix least-squares on *F*^2^ using SHELXL2014/4;^49^ these are included in the package of programs, Olex2.^50^ All non-hydrogen atoms were refined with anisotropic displacement parameters. Hydrogen atoms were placed in calculated positions and treated by a mixture of independent and constrained refinements with *U*_iso_(H) values set to 1.2*U*_eq_ of the attached carbon atoms and 1.5*U*_eq_ for methyl hydrogen atoms (CCDC 2305435-2305436). Crystallographic data are summarized in Table 1.

**Table 1:** Crystal Data and Refinement Parameters for **1** and **2**

crystal data	**1**	**2**
chemical formula	[Cu_2_(Hbdea)_2_Ni(CN)_4_]_*n*_	[Cu_2_(dmea)_2_Ni(CN)_4_]_*n*_·*n*H_2_O
*M*_r_	610.34	484.14
crystal system, space group	monoclinic, *P*2_1_/*c*	orthorhombic, *Pbca*
temp (K)	100	273
*a*, *b*, *c* (Å)	10.7057(8), 10.4530(2), 18.1790(13)	11.8276(5), 10.6309(4), 15.1649(5)
β (deg)	139.842(15)	90
*V* (Å^3^)	1311.9(3)	1906.80(12)
*Z*	2	4
radiation type	Cu Kα	Mo Kα
μ (mm^–1^)	3.02	3.21
crystal size (mm)	0.10 × 0.08 × 0.07	0.15 × 0.08 × 0.06
*T*_min_, *T*_max_	0.806, 0.981	0.629, 0.748
no. of measured, independent, observed [*I* > 2σ(*I*)] reflections	14592, 2557, 2327	34965, 1940, 1721
*R*_int_	0.036	0.048
(sin θ/λ)_max_ (Å^–1^)	0.621	0.625
*R* [*F*^2^ > 2σ(*F*^2^)], *wR*(*F*^2^), *S*	0.049, 0.164, 1.07	0.022, 0.055, 1.16
no. of reflections	2557	1940
no. of parameters	156	136
Δρ_max_, Δρ_min_ (e Å^–3^)	1.00, −0.78	0.29, −0.33

### 2.3 Magnetic Properties

Magnetic susceptibility measurements on crystalline samples of **1** and **2** were performed using a 6.5 T S700X SQUID magnetometer (Cryogenic Ltd.) over the temperature range from 4 to 300 K under an external magnetic field of 0.5 T. Polycrystalline samples (∼15 mg) were prepared and fixed in gelatin capsules. Each raw data file for the experimental magnetic susceptibility was corrected for the contributions of the sample holder, and the diamagnetic components of the constituent atoms were estimated from Pascal’s constants as −301.4 × 10^–6^ and −227 × 10^–6^ emu/mol for **1** and **2**, respectively.

### 2.4 Broken-Symmetry DFT Studies

The ORCA 5.0.4 package^51,52^ was used for all calculations. Unless stated otherwise, the B3LYP functional^53,54^ with the ma-def2-TZVPP basis set^55,56^ was used for all atoms for broken symmetry calculations. For single point and broken symmetry calculations, the SCF optimization convergence criteria were settled with the VeryTightSCF keyword, and integration grids of high density (Defgrid3 keyword) were employed. Optimization of geometries was performed using the ma-def2-SVP (preliminary calculations for H atoms optimization) or ma-def2-TZVP (final calculations) basis sets^55,56^ for all atoms (default SCF convergence criteria and default integration grids were applied). The CASSCF studies were performed using the def2-TZVPP basis set for copper atoms and their first coordination sphere and def2-SVP basis set^56^ for all other atoms. The AutoAux keyword^57^ was used to generate other auxiliary basis sets in all cases. The C-PCM solvation model^58^ with infinite dielectric constant was used as a rough model of a crystal field. The dispersion correction was introduced through the D4 model.^59,60^ Visualization of the molecular orbitals was performed by means of the Avogadro 1.2 program.^61,62^ Unless stated otherwise, the isosurfaces of the molecular orbitals are shown at the 0.02 au level. Extraction of exchange coupling constants was done through the equation *J* = −(*E*_HS_ – *E*_BS_)/(⟨**S**^2^⟩_HS_ – ⟨**S**^2^⟩_BS_).^63^ Analysis of bond critical points and noncovalent interactions indexes based on reduced density gradients (RDG)^64^ was performed using the Multiwfn 3.8 program.^65^ Selected ORCA outputs and Cartesian coordinates of the structural fragments used for the calculations are provided in Listings S1 and S5 (Supporting Information).

## 3 Results and Discussion

### 3.1 Structural Features

**Figure 1:**
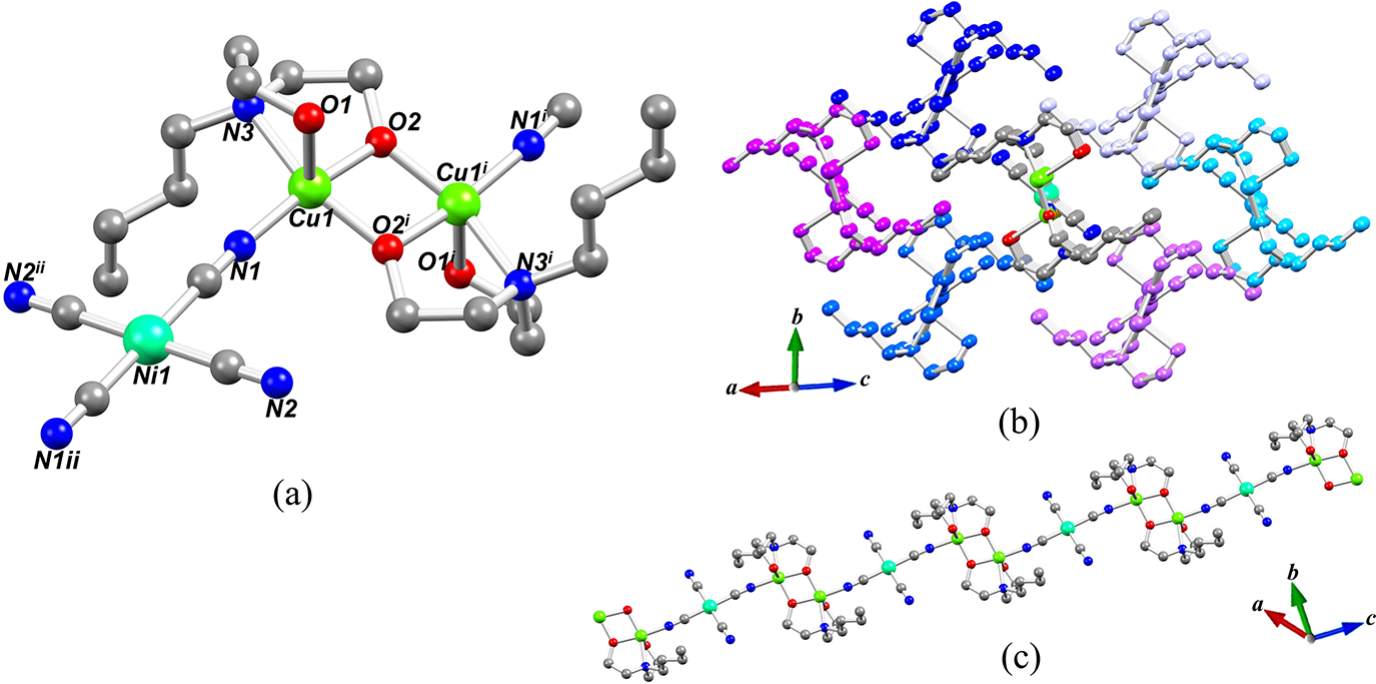
Crystal structure of **1**. (a) Representation of the [Cu_2_(Hbdea)_2_Ni(CN)_4_] unit. Symmetry codes: (i) 1 – *x*, 1 – *y*, 1 – *z* and (ii) −*x*, 1 – *y*, −*z*. (b) Crystal packing diagram showing a “central” 1D chain surrounded by six additional chains represented by different colors. (c) 1D coordination polymer chain made by alternating [Ni(CN)_4_]^2–^ and [Cu_2_(Hbdea)_2_]^2+^ units. (a–c) H atoms are not shown for clarity.

Both coordination polymers [Cu_2_(Hbdea)_2_Ni(CN)_4_]_*n*_ (**1**) and [Cu_2_(dmea)_2_Ni(CN)_4_]_*n*_·*n*H_2_O (**2**) bear the resembling dicopper(II) aminoalcoholate blocks and tetracyanonickelate linkers. The structural details are given in Table 1, while the relevant bonding parameters are listed in Table S1. The structure of **1** is composed of one-dimensional chains, running along the diagonal of the crystallographic *ac* plane. These 1D chains are assembled from the [Cu_2_(Hbdea)_2_]^2+^ units and [Ni(CN)_4_]^2–^ linkers (Figure 1), so that the Ni atoms are 4-coordinate and have an almost ideal square-planar geometry. As expected for a strongly bound ligand (C≡N), the Ni–C1 and Ni–C2 distances and the C1–Ni–C2 angle are 1.874(4) Å, 1.879(4) Å, and 88.73(17)°, respectively. The copper(II) centers in the dinuclear blocks are 5-coordinate and adopt a distorted square-pyramidal geometry. The Cu–O distances range from 1.907(3) to 2.338(3) Å, while the Cu–N bonds vary from 1.953(3) to 2.078(3) Å. The formation of a cyanide bridge can also be confirmed by FTIR spectroscopy. The geometry parameters are similar to those found in related coordination polymers.^34,66−69^

The geometric index of distortion for a nickel center is equal to τ_4_ = 0 (τ_4_′ = 0, square-planar), whereas for the copper centers the corresponding index is equal to τ_5_ = 0.21 [β = 171.16(13)°, α = 158.79(12)°], corresponding to a distorted square pyramid.^70,71^ Each resultant 1D chain features a 2C1 topology (Figure S6) and is surrounded by six additional chains with a spacing of 8.71–10.70 Å, based on the distances between the adjacent Ni atoms (Figure 1).

**Figure 2:**
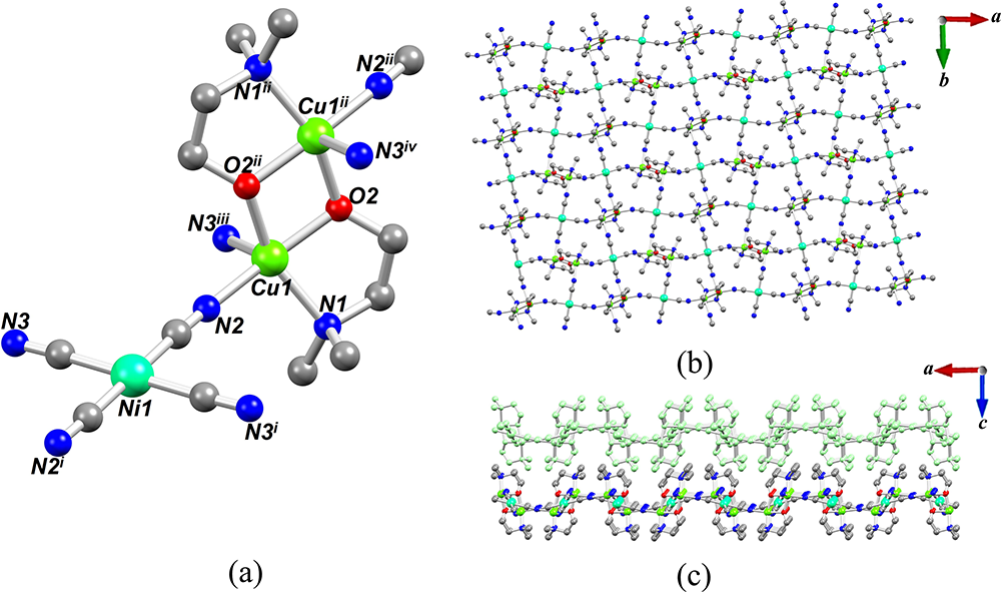
Crystal structure of **2**. (a) Representation of [Cu_2_(dmea)_2_Ni(CN)_4_] unit. Symmetry codes: (i) 2 – *x*, 1 – *y*, 1 – *z*, (ii) 1 – *x*, 1 – *y*, 1 – *z*, (iii) −1/2 + *x*, 1/2 – *y*, 1 – *z*, (iv) 3/2 – *x*, 1/2 + *y*, *z*. (b) 2D layers along the crystallographic *ab* plane. (c) View of stacked 2D layers along the *b*-axis. (a–c) H atoms are not shown for clarity.

As in the case of **1**, the crystal structure of **2** consists of the repeating dicopper(II) blocks [Cu_2_(μ-dmea)_2_]^2+^ and [Ni(CN)_4_]^2–^ linkers. However, in contrast to **1**, [Ni(CN)_4_]^2–^ acts as a 4-connected linker between four different dicopper(II) blocks, thus resulting in a two-dimensional coordination polymer structure. From a topological perspective (Figure S7), these layers can be described as a 4-connected uninodal net with a *sql* topology and a point symbol of (4^4^.6^2^).^16,72^ The 2D layers are stacked approximately 8 Å apart along the *c*-axis (Figure 2). The nickel atoms in **2** exhibit an almost ideal square-planar geometry (τ_4_ and τ_4_′ = 0), whereas the copper centers reveal a distorted square-pyramidal environment (τ_5_ = 0.17 to β = 163.97(9)° and α = 153.50(8)°). The M–O and M–N bond distances are comparable between **1** and **2** (Table S2). The Cu···Cu separation within dicopper units is very similar, namely, 3.0206(10) and 3.0060(5) Å for **1** and **2**, respectively. Powder X-ray diffractograms of **1** and **2** (Figures S3 and S4) agree with the calculated patterns from single-crystal data, confirming the phase purity of the compounds.

### 3.2 Magnetic Properties

**Figure 3:**
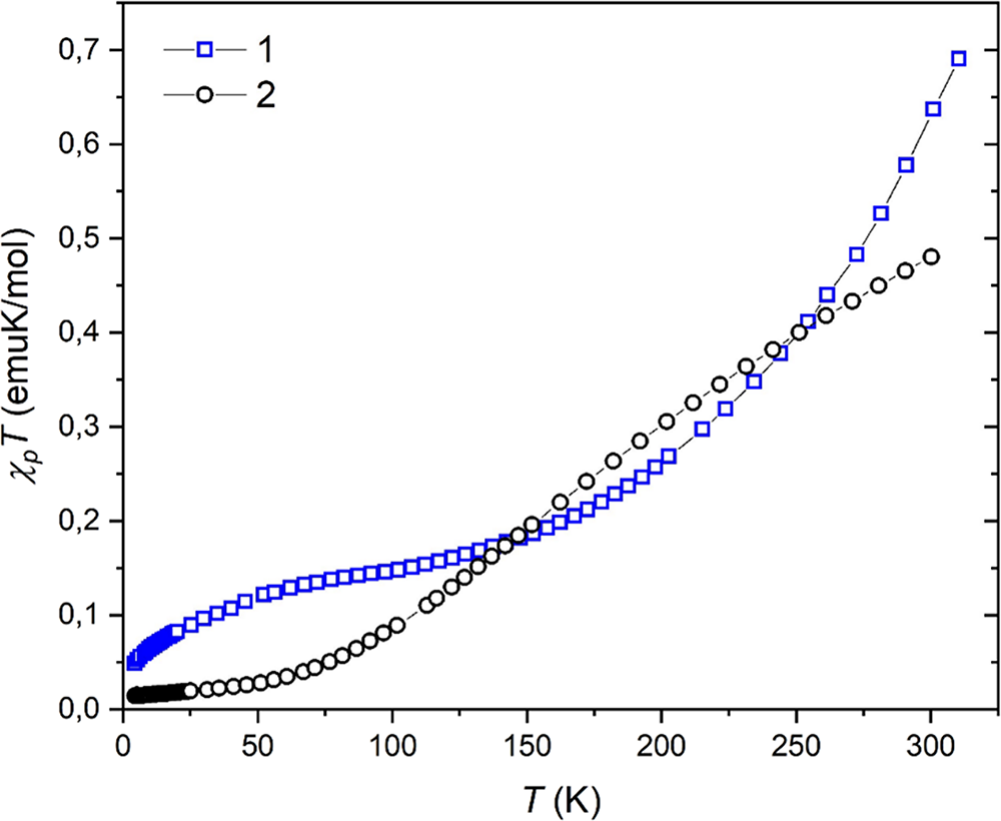
Temperature dependence of the χ_*p*_*T* product at 0.5 T for **1** (squares) and **2** (circles).

The magnetic properties of compounds **1** and **2** were measured on polycrystalline samples, the phase purity of which was confirmed by powder X-ray diffraction (Figures S3 and S4). As previously discussed, both compounds are made by alternating μ-O-alkoxo-bridged copper(II) dimers interlinked by diamagnetic [Ni(CN)_4_]^2–^. As a result, it is expected that the intradimer Cu(II)–Cu(II) interactions would dominate the magnetic behavior through magnetic coupling involving μ-O-alkoxo bridges. In both compounds the interdinuclear Cu···Cu distances are large (∼9 Å), suggesting that the contribution of the cyanide bridging ligand to the magnetic coupling is negligible. For **1**, the temperature dependence of the product of magnetic susceptibility (χ_*p*_) and temperature, χ_*p*_*T*, referred to a copper dimer unit, is shown in Figure 3. At room temperature (*T* = 301 K), χ_*p*_*T* is 0.62 emu K mol^–1^ per dimer, which is lower than the spin-only value of 0.75 emu K mol^–1^ for two *S* = 1/2 uncoupled Cu(II) ions (eq 1), indicating the occurrence of magnetic exchange. Here, *N* is Avogadro’s number, β refers to the Bohr magneton, *g* is the Landé factor with value *g* = 2, and *k* stands for the Boltzmann constant. As the temperature decreases, χ_*p*_*T* rapidly decreases from 300 to about 170 K, and below this decrease it becomes slower until approximately 50 K.
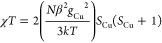
1This kind of magnetic behavior is consistent with the presence of a significant antiferromagnetic coupling between the two oxygen-bridged Cu(II) ions.^37,45,73−76^ In fact, the [Ni(CN)_4_]^2–^ linkers can only provide very weak magnetic interactions (see the Theoretical Calculations section), so strong antiferromagnetic coupling is predicted to be mediated by the μ-O-alkoxo bridges.

For **2**, the temperature dependence of the χ_*p*_*T* product (Figure 3) only reaches 0.48 emu K mol^–1^ at room temperature (*T* = 300 K), which is much lower than the expected value for a system of two independent Cu(II) ions (*S* = 1/2), considering *g* = 2.00. As the temperature decreases, the system shows a decrease of χ_*p*_*T* to about 70 K at a slower pace compared to **1**. Below this, χ_*p*_*T* smoothly decreases as the temperature goes down, reaching almost zero at 4 K, denoting the presence of an overall strong antiferromagnetic behavior. Furthermore, the fact that the high-temperature χ_*p*_*T* limit was not yet achieved at 300 K for compounds **1** and **2** is consistent with the presence of strong exchange couplings within the dicopper(II) units. Therefore, and similarly to **1**, it is assumed that **2** behaves as an antiferromagnetically coupled dicopper(II) system where the [Ni(CN)_4_]^2–^ units do not play any significant role.

**Figure 4:**
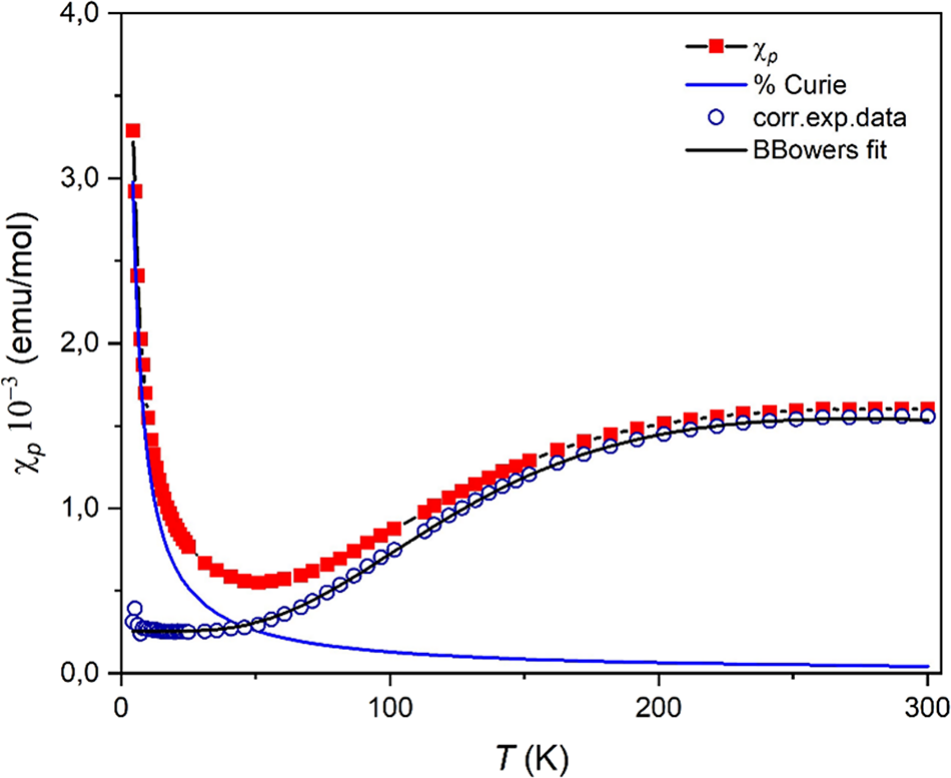
Temperature dependence of the χ_*p*_ product at 0.5 T for **2**. The black solid line represents the fit according to the Bleaney–Bowers model (eq 2) with *J* = −151 cm^–1^ (see text).

From the plot of χ_*p*_ as a function of temperature (Figure 4) for **2**, a clear behavior for a Cu dimer can be observed with a maximum near 280 K. At very low temperatures, a rapid increase of χ_*p*_ as a commonly observed Curie tail for coupled antiferromagnetic systems indicates a small percentage of paramagnetic impurities. Accordingly, an equation considering three terms including a major contribution of two antiferromagnetically coupled *S* = 1/2 spins was proposed to analyze the temperature dependence of the magnetic susceptibility of **2** (eq 2). It is described by the Bleaney–Bowers equation^75^ and based on the model of an isolated Heisenberg dimer of *S* = 1/2 ions with Hamiltonian interaction *H* = −2*JS*_1_*S*_2_ (where *S*_1_ = *S*_2_ for Cu(II)), a temperature-independent paramagnetism (TIP) term, and a low-temperature Curie contribution (eq 2). Herein, *J* is the intradimer superexchange interaction parameter between two Cu(II) ions, ρ is the parameter accounting for the percentage of paramagnetic impurity, *C* is the Curie constant, and θ is a Weiss temperature correction.
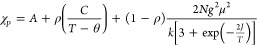
2As shown in Figure 4, the determined fitting results are in very good agreement with the experimental data giving the best-fit parameters *J* = −151.0 cm^–1^ and *g* = 2.08 with a Curie tail corresponding to 2.8% of paramagnetic impurities and a TIP term *A* of 7.8 × 10^–4^. The negative *J* value indicates the existence of antiferromagnetic interactions occurring between the two Cu(II) ions, as supported by the shape of the χ_*p*_*T*–*T* plot. The obtained *g* value is reasonable for Cu(II).

**Figure 5:**
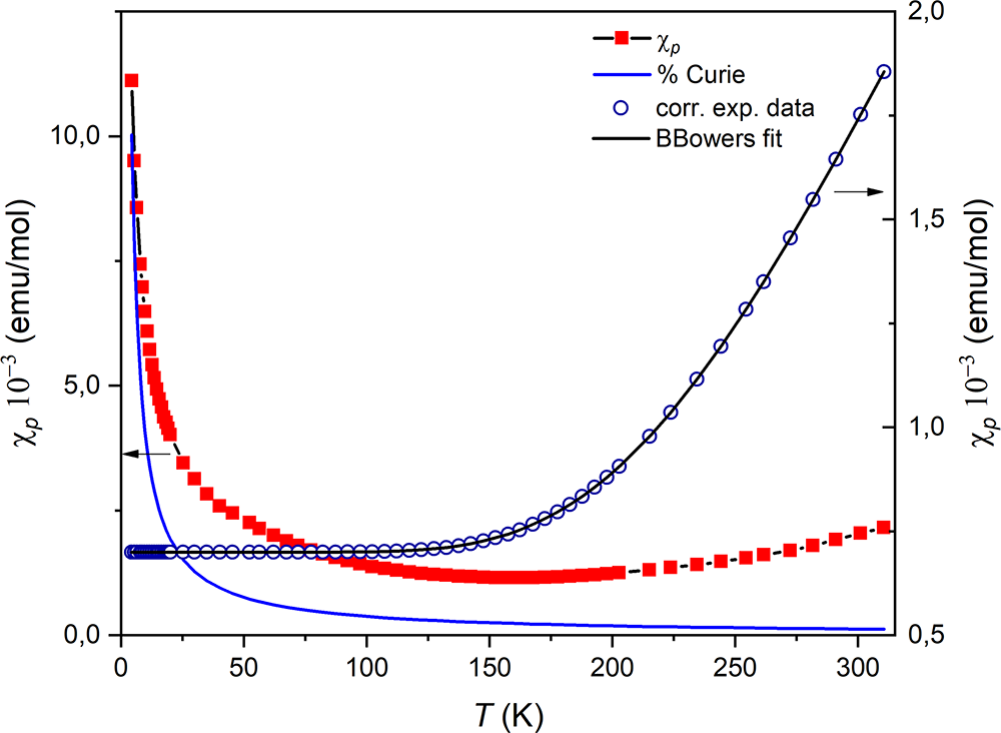
Temperature dependence of the χ_*p*_*T* product for **1**. The solid line represents a fit with the Bleaney–Bowers model (eq 2) with *J* = −602.1 cm^–1^ (see text).

By performing the same type of analysis for **1** (Figure 5), the absence of a maximum in χ_*p*_ vs *T* in the range of measured temperatures is noted, suggesting that the antiferromagnetic interaction coupling constant (*J*) may be considerably larger than the exchange constant for **2**. In fact, fitting eq 2 to the experimental data resulted in *J* = −602.1 cm^–1^ with *g* = 2.32, 4.3% of paramagnetic impurities, and a TIP contribution of 7.0 × 10^–4^. In fact, the exchange coupling within the dimer is stronger in this case compared to **2**, confirming the presence of stronger antiferromagnetic interactions.

The strong antiferromagnetic behavior and the observed differences in *J* values are related to different geometric parameters around the copper(II) centers within the dimeric units. For hydroxo- and alkoxo-bridged copper(II) compounds, the magnetic properties are influenced by the intradimer angles (Cu–O–Cu) as well as the hinge distortion of the [Cu_2_(μ-O)_2_]^2+^ core or the out-of-plane shift of the carbon atom from the alkoxo bridge.^44,73,77^ In fact, magneto structural studies demonstrated that in the Cu dimers showing a superexchange between the paramagnetic centers through the bridging alkoxide O atoms, the value of Cu–O–Cu bridging angle (θ) is the primary geometric factor affecting the *J* value and the type of magnetic exchange interaction: the larger the angle, the stronger the antiferromagnetic coupling between the Cu(II) ions.^78−81^ An antiferromagnetic coupling dominates for θ higher than 98°, while a ferromagnetic interaction is predominant for θ lower than 98°. Compounds **1** and **2** present Cu–O–Cu angles of 104.09(12)° and 101.80(7)°, respectively, which are in agreement with the correlations established for alkoxo-, carboxylate-, and hydroxo-bridged Cu(II) structures. For **1**, a stronger antiferromagnetic coupling (*J* = −602.1 cm^–1^) is in line with a higher value of θ.

### 3.3 Theoretical Calculations

The broken symmetry concept at the DFT level provides a fast and useful mechanism for the evaluation of magnitude of the magnetic exchange between spin carriers.^82−84^ Although this approach suffers from several drawbacks, such as a strong dependency on the DFT functional and necessity of the spin decontamination procedure of the broken symmetry state, its straightforward nature makes it an important instrument in modern computational chemistry. In the present study the B3LYP hybrid functional was chosen as it is known to generate the reliable values of exchange couplings in a wide range of systems.^85−87^

**Figure 6:**
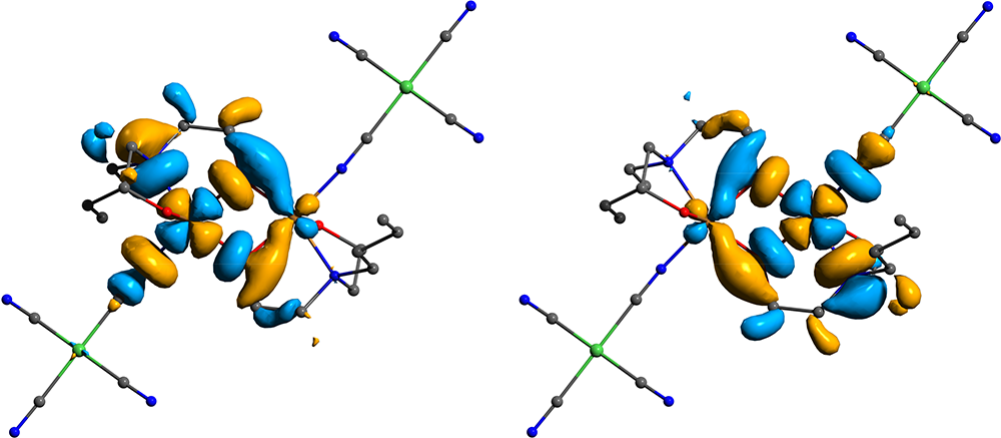
Isosurfaces of the unrestricted corresponding magnetic orbitals for **1a** obtained from B3LYP/ma-def2-TZVPP broken symmetry calculations. H atoms are omitted for clarity. The {Cu(μ-O)_2_Cu} core is aligned in the *xy* plane.

**Figure 7:**
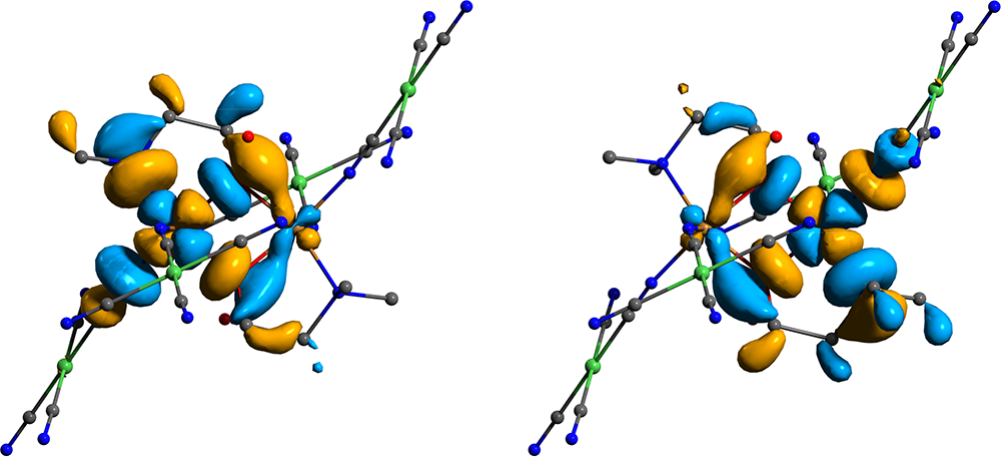
Isosurfaces of the unrestricted corresponding magnetic orbitals for **2a** obtained from B3LYP/ma-def2-TZVPP broken symmetry calculations. H atoms are omitted for clarity. The {Cu(μ-O)_2_Cu} core is aligned in the *xy* plane.

Since the solid-state crystal structures of **1** and **2** constitute coordination polymers, the selection of a proper 0D fragment of a structure is necessary prior to modeling the magnetic exchange. The structures feature dinuclear copper units where each copper atom is coordinated by one (**1**) or two (**2**) tetracyanonickelate blocks (Figures 1 and 2). Also, the structure of **2** contains water molecules that are located close to the ligand O-sites presumed to participate in the magnetic superexchange pathway. From these assumptions, the [Cu_2_(Hbdea)_2_{Ni(CN)_4_}_4_]^2–^ (**1a**) and {[Cu_2_(dmea)_2_{Ni(CN)}_4_](H_2_O)_2_}^6–^ (**2a**) fragments were selected as starting points. The tetracyanonickelate block is expected to be in the diamagnetic low-spin state,^88,89^ which agrees with the experimental measurements of magnetic susceptibility of **1** and **2**. The broken symmetry calculations at the B3LYP/ma-def2-TZVPP level of theory suggested a strong antiferromagnetic coupling in both **1a** and **2a**, with the corresponding magnetic orbitals overlap integral *S*_ab_ of 0.24941 and 0.17169, respectively (Figures 6 and 7). One can note the strong delocalization of these orbitals. The large overlap suggested the use of a spin-projected method by Yamaguchi et al.^63^ to extract the final *J* values (see the Methods section). The predicted singlet–triplet gap for **1a** is considerably higher than that for **2a** (−657.40 and −238.41 cm^–1^, respectively; the *Ĥ* = −2*JŜ*_1_*Ŝ*_2_ formalism was used). This follows the geometry of the {Cu(μ-O)_2_Cu} cores where the Cu–O–Cu angle for **1a** (103.9°) is slightly higher than that for **2a** (101.8°).^90^ This tendency follows the disposition of experimental values for **1** and **2**, where compound **1** shows a stronger exchange (Table 2). For comparative purposes, the broken symmetry calculations of fragments **1a** and **2a** were performed using the common DFT functionals of meta-GGA and hybrid meta-GGA classes: M06-L,^91^ r^2^SCAN,^92^ TPSSh,^93^ and PBE0^94^ (entries **a**^**I**^–**a**^**VI**^ in Table 2). As can be seen, all of the functionals stabilize the broken symmetry state and significantly overestimate the singlet–triplet gap (Table 2) as compared to the experimental data and the B3LYP functional. The TPSSh functional performs best after the B3LYP one. Earlier, the TPSSh functional was shown to outperform B3LYP for manganese coupled systems;^95^ thus, for copper d^9^ compounds it is expected to have similar performance. Apart from the functional, the choice of a spin-decontamination scheme is an important step. The Yamaguchi et al.^62^ expression routinely applied in the present study covers a broad range of interaction magnitudes, from weak to strong ones. However, the expression by Bencini, Noodleman, and Ruiz (BNR) et al.^96−98^*J* = −(*E*_HS_ – *E*_BS_)/(**S**_max_(*S*_max_ + 1)) results in a significant decrease of the predicted singlet–triplet gaps up to almost quantitative prediction of *J* constants for both **1a** and **2a** in the case of the M06-L functional (Table 2, **a**^**III**^). Considering a large scattering of couplings predicted at different levels, the outstanding results demonstrated by the M06-L/BNR-expression level are likely a coincidence than a systematic result. It should be noted that the 2 kcal mol^–1^ precision can be viewed as excellent for DFT methods, while it corresponds to 700 cm^–1^—a value higher than the magnitudes of typical exchange couplings. Thus, the broken symmetry calculations presented here describe the magnitudes of exchange couplings with a reasonable deviation from the experimentally determined values.

**Table 2:** Experimental and DFT Calculated Exchange Coupling Constants^a^

	complex		
	**1**	**2**	notes
	–602.1	–151.0	experimentally measured
**a**	–657.40	–238.41	all crystallographic coordinates
**a^I^**	+698.86	+245.62	unprojected *E*_HS_–*E*_BS_ gap
**a^II^**	–1050.96	–366.04	M06L
**a^III^**	–623.20	–198.79	M06L with *J* = −(*E*_HS_ – *E*_BS_)/(*S*_max_(*S*_max_ + 1)) spin projection scheme
**a^IV^**	–869.41	–334.88	TPSSh
**a^V^**	–978.85	–343.25	R^2^SCAN
**a^VI^**	–1460.38	–596.03	PBE0
**b**	–661.30	–249.78	positions of H atoms optimized^b^
**c**	–670.37	–234.77	added protons to all Ni–CN groups^c^
**d**		–267.68	removed water molecules^d^
**e**	–664.34		added protons only to certain Ni–CN groups^e^
**f**	–666.17		added protons only to certain Ni–CN groups and truncated ligands^f^
**g**	–657.27		ligands truncated^g^
**h**	–679.19		HCN–Cu instead of [Ni(CN)_4_]–Cu^h^
**i**	–670.53		CH_3_CN–Cu instead of [Ni(CN)_4_]–Cu^i^

a Unless stated otherwise, the constants were calculated at the B3LYP/ma-def2-TZVPP level using *Ĥ* = −2*JŜ*_1_*Ŝ*_2_ spin Hamiltonian. The H atoms positions were optimized in all cases where protons were added or ligands transformed.

b Optimization was performed at the B3LYP/ma-def2-TZVP/D4 level.

c 6 and 12 protons were added to the models **1b** and **1c**, respectively.

d The water molecules were eliminated from the model **2b** without further optimizations.

e two protons added to the Ni–CN groups in the *trans*-position relative to copper centers.

f The same as footnote *e* but with truncation of *n*-butyl groups to methyl ones. The def2-TZVPP basis set was used for copper atoms and first coordination sphere (including C atom of the cyanido groups), and def2-SVP was used for all remaining atoms.

g Truncation of *n*-butyl groups to methyl ones.

h Protons added instead of Ni(CN)_3_ groups.

i Methyl groups were added instead of Ni(CN)_3_ ones with optimization of positions of all H atoms and the C atom of methyl groups.

**Figure 8:**
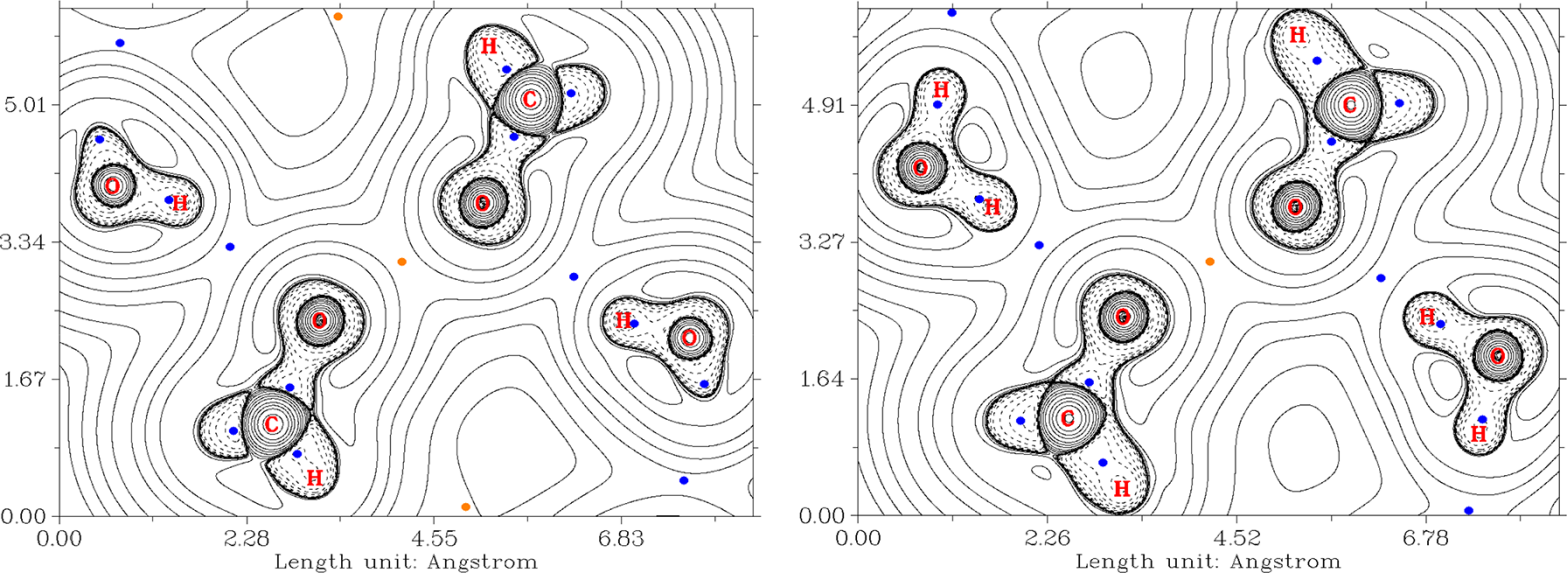
Plots of the Laplacian of the electron density in the real space, ∇^2^ρ(**r**), showing the O–H···O hydrogen bonds between the uncoordinated water molecules and bridging oxygen atoms of the {Cu(μ-O)_2_Cu} core before (left, model **1a**) and after (right, model **1b**) optimization of H atoms’ positions.

**Figure 9:**
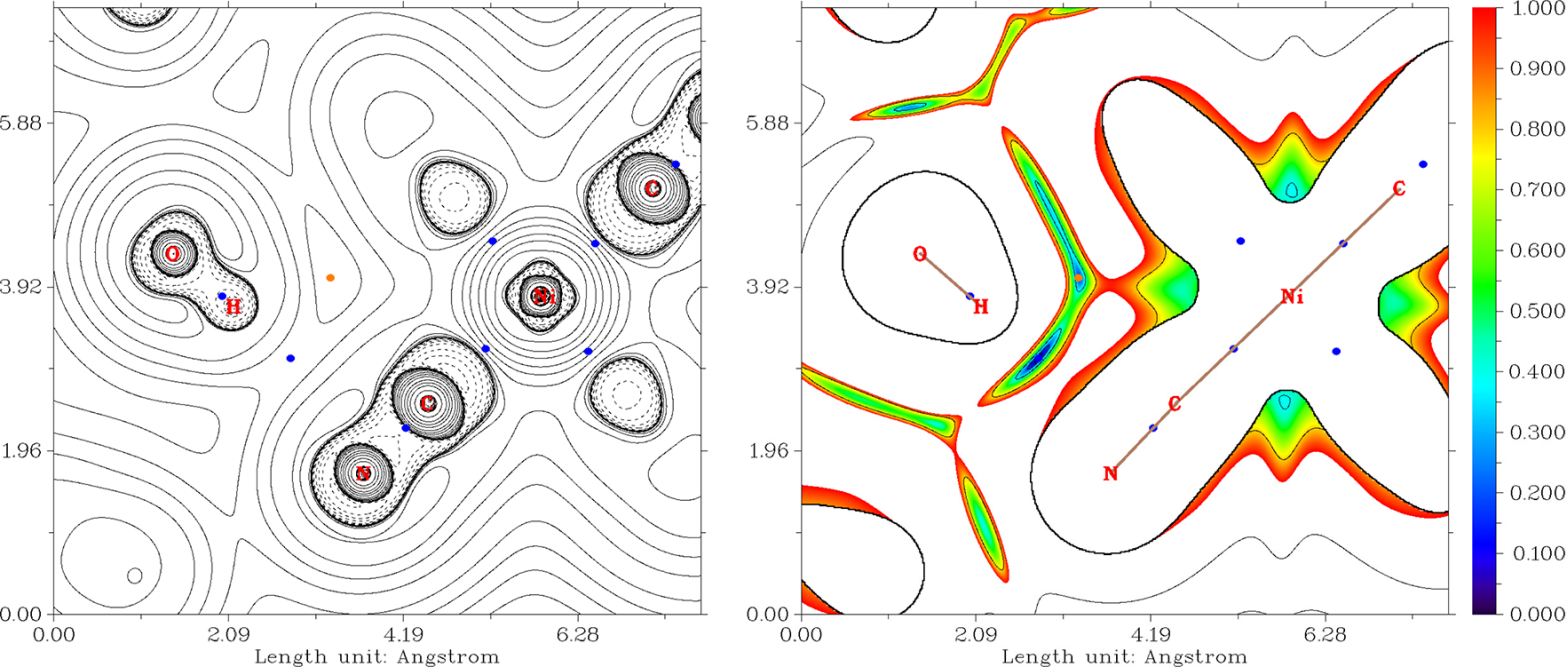
Left: plot of the Laplacian of the electron density in the real space, ∇^2^ρ(**r**), showing the hydrogen bond between the uncoordinated water molecules and cyanido group after optimization of the positions of H atoms in **1b**. Right: plot of the RDG function for the same projection. The color scheme corresponds to the sign(λ_2_)ρ(**r**) function with the second largest eigenvalue of the Hessian of electron density, λ_2_, at the respective points.

At the next step, we investigated the influence of structural and charge corrections on the magnitude of magnetic exchange. It is known that the coordinates of hydrogen atoms are poorly determined from the X-ray crystallographic data, where most often the X–H distances and angles are constrained due to a low scattering factor of hydrogen atoms. The respective constraints, however, not always correspond to the real geometry,^99,100^ especially in the case of O–H groups. Hence, optimization of the H atom positions in the fragment may change the predicted magnitude of magnetic coupling. Positions of the H atoms in fragments **1a** and **2a** were optimized at the B3LYP/ma-def2-TZVP/D4 level, resulting in the expected elongation of all X–H bonds (fragments **1b** and **2b**). In the case of **2b**, the hydrogen atoms of the water molecule underwent a rotation, enforcing the O–H···O hydrogen bond with the alkoxy group (Figure 8) and making a weak hydrogen bond O–H···NC with the cyanido group, as evidenced by the Laplacian as well as reduced density gradient (RDG)^64^ plots (Figure 9). The electron densities in the respective bond critical points ρ(**r**_BCP_) constitute 2.17 × 10^–2^ and 8.8 × 10^–3^ a.u., respectively. According to the relation proposed by Emamian et al.,^101^ these densities correspond to the binding energies of −34.7 and −16.7 kJ mol^–1^, respectively. Thus, considering these hydrogen bonds only, the binding energy of a water molecule to a metal-complex framework can be considered as no less than −51.4 kJ mol^–1^. The direct calculations of the binding energy (BE) at the B3LYP/ma-def2-TZVPP/D4 level resulted in the twice lower energy of −27.4 kJ mol^–1^ per water molecule (where BE_AB_ = *E*_AB_ – *E*_A_ – *E*_B_). This may account for the limited applicability of the general empirical BE vs ρ(**r**_BCP_) equations and necessity of training the coefficients of these equations for each particular case, as was stated earlier.^102−104^

The broken symmetry calculations of the hydrogen-optimized fragments **1b** and **2b** revealed the resembling *J* values compared to those obtained for **1a** and **2a** (Table 2). Moreover, further alterations of the structure by adding protons to simulate coordination of the cyanido ligands and compensate negative charge, as well as truncating the *n*-butyl groups for **2** or replacing its tetracyanonickelate blocks with HCN and CH_3_CN, affected the magnitude of the predicted exchange coupling to a very limited extent (Table 2), keeping the *J* values calculated for the full crystallographic coordinates **1a** and **2a** most close to those determined experimentally.

The possibility of [Ni(CN)_4_]^2–^ block to transmit superexchange interactions was studied by constructing a model fragment {[Cu(H_2_bdea)(H_2_O)][Ni(CN)_4_][Cu(H_2_bdea)(H_2_O)]}^2+^, where the water molecules were generated by using the coordinates of the respective alkoxy-bridging atoms of the ligand (Figure S8). Only a very weak antiferromagnetic coupling of −0.33 cm^–1^ was predicted (magnetic orbital overlap *S*_ab_ = 0.00447) in this way, suggesting that the dinuclear copper blocks are rather magnetically isolated.

According to the single point open-shell DFT calculations at the B3LYP/ma-def2-TZVPP level, the unpaired electrons are located at the d_*x*^2^–*y*^2^_ copper orbitals in all studied cases, as expected from the square-pyramidal coordination geometries around the copper centers. The fragment **1f** containing two protons at the terminal cyanido groups and truncated *n*-butyl groups was used for further comparative studies. The broken symmetry calculations using the B3LYP functional and ma-def2-TZVPP basis set for all atoms reveal *J* = −657.27 cm^–1^ (Table 2). Because of the absence of a negative charge in **1f**, the diffuse function of the basis sets becomes not necessary and can be excluded. Further, a small involvement of [Ni(CN)_4_] blocks into the magnetic exchange suggests the possibility of using the large basis set on the copper atoms and their nearest surroundings only. Thus, the calculations using the def2-TZVPP basis set for copper and the first coordination sphere, keeping all other atoms at the def2-SVP level, resulted in *J* = −666.17 cm^–1^, a value that is very close to all others calculated for **1** (Table 2). The corresponding magnetic orbitals of **1f** (Figure S9) are of 58.5% of d_*x*^2^–*y*^2^_ character and involve 5.6% and 3.3% of p_*x*_ and p_*y*_ atomic orbitals of bridging oxygen atoms for each of the α and β magnetic orbitals. The restricted open shell (ROKS) calculations resulted in the singly occupied frontier molecular orbitals (SOMO) of shape similar to that of unrestricted corresponding orbitals (Figure 9), which contain 64% and 54% of d_*x*^2^–*y*^2^_ copper atomic orbitals, the latter involving 8.6 and 10.2% of the oxygens of p_*x*_ and p_*y*_ atomic orbitals, respectively, showing some degree of delocalization over the bridging atoms. The energy gap between the ROKS/SOMO orbitals is 9622 cm^–1^.

The state-averaged SA-CASSCF calculations for the **1f** and **2c** models in the smallest possible (2,2) active space resulted in the ferromagnetic ground state with the first singlet 209.3 and 256.9 cm^–1^ above, respectively. The active space orbitals are of the same shape as the ROKS/SOMO and localized orbitals and are of d_*x*^2^–*y*^2^_ character as well (Figure S9). Although in this case the singlet–triplet gap is smaller for the model of complex **1**, which follows the experimental tendency (Table 2), the inverse ground state accounts for incorrect energies of the singlet–triplet splittings obtained for the very limited CAS(2,2) model. The involvement of all copper d-orbitals into the active space changes the energy gap drastically, predicting the correct antiferromagnetic ground state for CAS(18,10) for **1f** with the first triplet state is just 44 cm^–1^ above that. Such an underestimation is known for pure multireference HF, where the lack of electron correlation prevents quantitative prediction of the exchange couplings. Although the multireference PT methods can partially recover the correlation energy, the true correspondence of theoretical and experimental exchange coupling in the multireference framework can be achieved only by using the DDCI3 methods that involve excitations out of the active space (CAS).^105,106^ Considering a little expected improvement from NEVPT2 and related methods, as well as extraordinary computational resources required by the DDCI ones, we have limited our studies to a pure CAS(18,10) model of **1f**.

**Figure 10:**
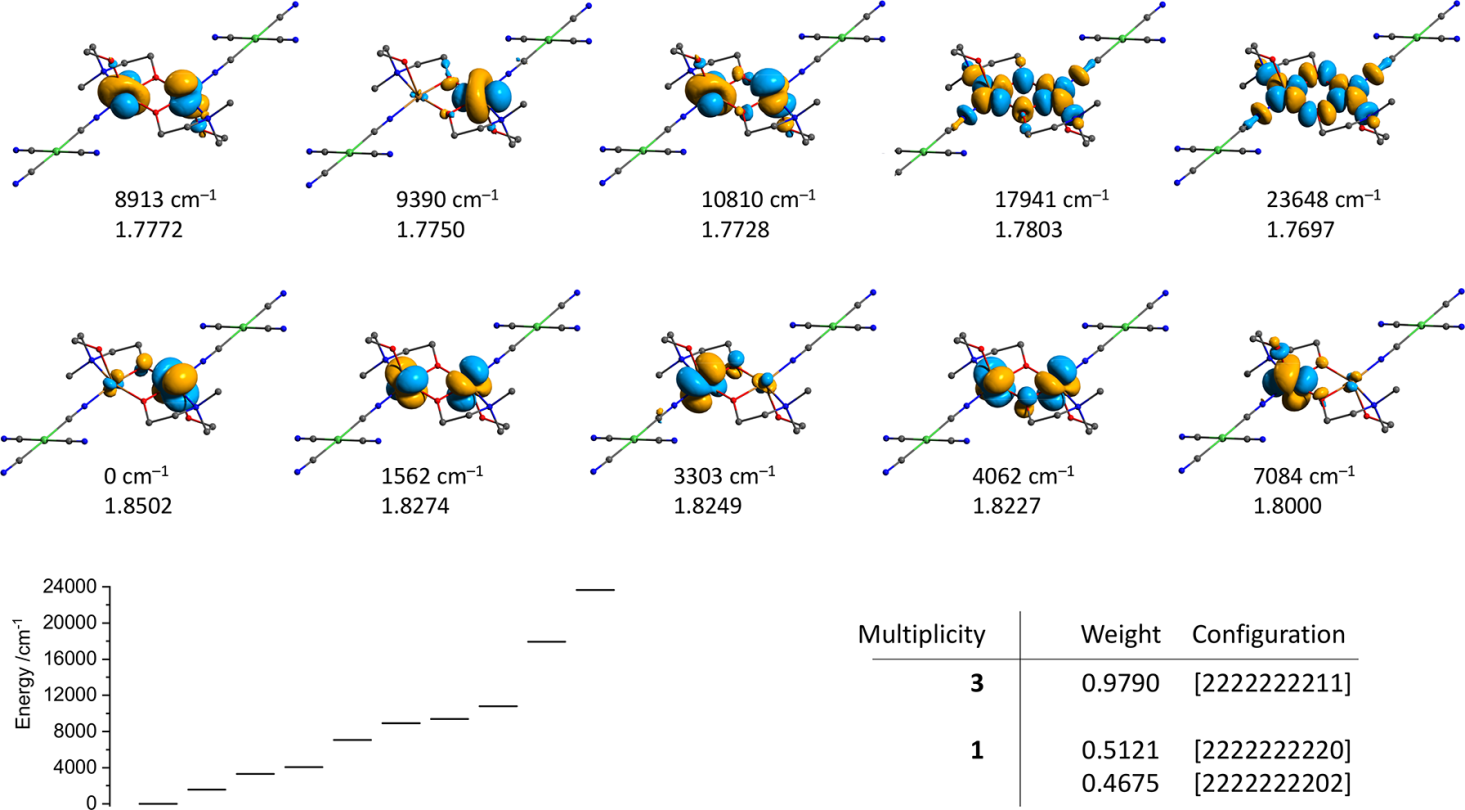
Top: isosurfaces of the molecular orbitals of the CAS(18,10) active space for **1f** with the relative energies and populations (25 and 20 roots were considered for singlet and triplet states, respectively). H atoms are omitted for clarity. Left bottom: the diagram showing the relative energies of the above orbitals in the same order. Right bottom: largest configuration contributions to the lowest singlet and triplet states. The def2-TZVPP basis set was used for copper atoms and first coordination sphere (including the carbon atoms of Cu–CN groups) and the def2-SVP basis set for all other atoms.

The ten orbitals and their relative energies obtained by the SA-CAS(18,10) calculations for **1f** are depicted in Figure 10. The orbitals, corresponding to the magnetic ones (Figure S9), are those having the highest energies and containing 73.4 and 71% d_*x*^2^–*y*^2^_ atomic orbitals. The respective energy gap is 5707 cm^–1^ (Figure 10), being close to that predicted by the DFT/ROKS calculations (9622 cm^–1^). While the first ferromagnetic state was always found have a single configuration in all cases (>97% of the [11] configuration for the highest orbitals, Figure 10), the antiferromagnetic ground spin state is always constructed of an approximately equal mixture of [20] and [02] determinants. This contrasts significantly with the UHF DFT broken symmetry model that describes this spin state as a single reference one, where unpaired electrons located on copper centers have opposite spins. The state-specific CAS(18,10) calculation for singlet and triplet states of **1f** revealed a similar multireference nature of the singlet state (Listing S4), where the ground state is a singlet and the singlet–triplet gap is 60.7 cm^–1^.

## 4 Conclusions

In this study, we further explored a convenient self-assembly method for the generation of two new coordination polymers **1** and **2** in water. The obtained compounds are built from the dicopper(II)–aminoalcoholate blocks and the tetracyanonickelate(2−) linkers. Both coordination polymers were fully characterized by standard methods, and their structures were established by single-crystal X-ray diffraction. The latter revealed the formation of 1D chains in **1** and 2D metal–organic layers in **2**.

The magnetic behavior of CPs **1** and **2** was investigated in detail, and the obtained results indicate that both compounds display strong antiferromagnetic coupling (*J* = −602.1 cm^–1^ for **1** and −151.0 cm^–1^ for **2**) between Cu(II) ions within the dimers. The broken symmetry DFT calculations supported strong antiferromagnetic couplings for both CPs. A range of model fragments was studied, showing irrelevance of the predicted exchange on the positions of H atoms as well as the nature of the substituents. The CASSCF calculations confirmed the antiferromagnetic coupling within the {Cu(μ-O)_2_Cu} cores, where the singlet ground spin state has multireference character. Both DFT and CASSCF studies suggested that tetracyanonickelate blocks are not involved in the magnetic coupling between the copper centers nor act as superexchange bridges between dimeric blocks.

In summary, this work widens the growing family of cyanometalate-driven coordination polymers, showing that such compounds can be assembled from simple chemicals and by using self-assembly methods in water. Further research on extending the family of related heterodimetallic coordination polymers is currently underway in our laboratory.

## Supporting Information Available

Materials and instruments, images of compounds (Figure S1), FTIR-ATR spectra (Figure S2), PXRD patterns (Figures S3 and S4), TGA data (Figure S5), bonding parameters (Table S1), additional figures for **1** and **2** (Figures S1 and S6–S9), and DFT calculated Cartesian coordinates (Listings S1–S4) (PDF)

## References

[ref1] Yang J.; You M.-L.; Liu S.; Deng Y.-F.; Chang X.-Y.; Holmes S. M.; Zhang Y.-Z. Cyanide-Bridged Rope-like Chains Based on Trigonal-Bipyramidal [Fe2Cu3] Subunits. Inorg. Chem. 2023, 62, 17530–17536. 10.1021/acs.inorgchem.3c02986. 37801447

[ref2] Shukla P.; Das S.; Bag P.; Dey A. Magnetic Materials Based on Heterometallic Cr II/III – Ln III Complexes. Inorg. Chem. Front. 2023, 10, 4322–4357. 10.1039/D3QI00193H.

[ref3] Su F.; Li S.; Han C.; Wu L.; Wang Z. Tuning Three Coordination Polymers with Dinuclear Metal Units via PH Control: Syntheses, Structures, and Magnetic Properties. J. Solid State Chem. 2022, 311, 123121 10.1016/j.jssc.2022.123121.

[ref4] An Z. W.; Gao Y. Q.; Xu S. M.; Zhang W.; Yao M. X. 3d Ion-Driven Hexanuclear Heterometallic Clusters with Amazing Structures and Magnetic Properties. Cryst. Growth Des. 2023, 23, 1412–1421. 10.1021/acs.cgd.2c00940.

[ref5] Zhang Y.; Yang Q.; Lu J.; Guo M.; Li X.-L.; Tang J. Heterometallic {DyIII2FeII2} Grids with Slow Magnetic Relaxation and Spin Crossover. Inorg. Chem. Front. 2021, 8, 1779–1787. 10.1039/D0QI01471K.

[ref6] Yu S.; Zhang Q.-H.; Chen Z.; Zou H.-H.; Hu H.; Liu D.; Liang F.-P. Structure, Assembly Mechanism and Magnetic Properties of Heterometallic Dodecanuclear Nanoclusters DyIII4MII8 (M = Ni, Co). Inorg. Chem. Front. 2021, 8, 5214–5224. 10.1039/D1QI01051D.

[ref7] Mínguez Espallargas G.; Coronado E. Magnetic Functionalities in MOFs: From the Framework to the Pore. Chem. Soc. Rev. 2018, 47, 533–557. 10.1039/C7CS00653E. 29112210

[ref8] Falsaperna M.; Saines P. J. Development of Magnetocaloric Coordination Polymers for Low Temperature Cooling. Dalton Trans. 2022, 51, 3394–3410. 10.1039/D1DT04073A. 35106524

[ref9] Mwanza T.; Kürkçüoğlu G. S.; Ünver H.; Şahin O.; Yeşilel O. Z. Synthesis, Spectroscopic, Structural Characterizations, and Catalytic Properties of Cyanide-Bridged Heteronuclear Metal Organic Frameworks with Imidazole. J. Solid State Chem. 2022, 314, 123344 10.1016/j.jssc.2022.123344.

[ref10] Zakrzewski J. J.; Liberka M.; Zychowicz M.; Chorazy S. Diverse Physical Functionalities of Rare-Earth Hexacyanidometallate Frameworks and Their Molecular Analogues. Inorg. Chem. Front. 2021, 8, 452–483. 10.1039/D0QI01197E.

[ref11] Sun A.-H.; Liu X.-X.; Sun R.; Xiong J.; Sun H.-L.; Gao S. The Rational Construction of Diamond-like Dysprosium–Hexacyanometallate Frameworks Featuring Dynamic Magnetic Behaviour. Inorg. Chem. Front. 2022, 9, 231–240. 10.1039/D1QI01173A.

[ref12] Ahmed S.; Kumar A.; Mukhopadhyay N.; Lloret F.; Mukherjee R. Heterobimetallic Cyanide-Bridged FeIII(μ-CN)MII Complexes (M = Mn and Cu): Synthesis, Structure and Magnetism. New J. Chem. 2022, 46, 7711–7720. 10.1039/D2NJ00126H.

[ref13] Yang J.; Zhao X. H.; Deng Y. F.; Zhang X. Y.; Chang X. Y.; Zheng Z.; Zhang Y. Z. Azido-Cyanide Mixed-Bridged FeIII-NiIIComplexes. Inorg. Chem. 2020, 59, 16215–16224. 10.1021/acs.inorgchem.0c01917. 33105988

[ref14] Alexandru M. G.; Visinescu D.; Cano J.; Lloret F.; Julve M. Cyanido-Bridged Heterobimetallic Molecular Squares: Low-Dimensional Models of Prussian Blue Analogues and Beyond. Cryst. Growth Des. 2023, 23, 1288–1308. 10.1021/acs.cgd.2c01366.

[ref15] Yue Q.; Gao E. Q. Azide and Carboxylate as Simultaneous Coupler for Magnetic Coordination Polymers. Coord. Chem. Rev. 2019, 382, 1–31. 10.1016/j.ccr.2018.12.002.

[ref16] Kürkçüoğlu G. S.; Yeşilel O. Z.; Sayın E.; Enönlü E.; Şahin O. Synthesis and Structural Analysis of Heteronuclear Hexacyanochromate(III) Complex with Tris(2-Aminoethyl)Amine), [Cd(Tren)(Htren)][Cr(CN)6]·2H_2_O. J. Mol. Struct. 2020, 1219, 128462 10.1016/j.molstruc.2020.128462.

[ref17] Alexandrov E. V.; Virovets A. V.; Blatov V. A.; Peresypkina E. V. Topological Motifs in Cyanometallates: From Building Units to Three-Periodic Frameworks. Chem. Rev. 2015, 115, 12286–12319. 10.1021/acs.chemrev.5b00320. 26505277

[ref18] Avila Y.; Acevedo-Peña P.; Reguera L.; Reguera E. Recent Progress in Transition Metal Hexacyanometallates: From Structure to Properties and Functionality. Coord. Chem. Rev. 2022, 453, 214274 10.1016/j.ccr.2021.214274.

[ref19] Pavlik J.; Masárová P.; Nemec I.; Fuhr O.; Ruben M.; Šalitroš I. Heteronuclear Iron(III)-Schiff Base Complexes with the Hexacyanidocobaltate(III) Anion: On the Quest to Understand the Governing Factors of Spin Crossover. Inorg. Chem. 2020, 59, 2747–2757. 10.1021/acs.inorgchem.9b03097. 32045222

[ref20] Yanai N.; Kaneko W.; Yoneda K.; Ohba M.; Kitagawa S. Reversible Water-Induced Magnetic and Structural Conversion of a Flexible Microporous Ni(II)Fe(III) Ferromagnet. J. Am. Chem. Soc. 2007, 129, 3496–3497. 10.1021/ja069166b. 17341085

[ref21] Shatruk M.; Dragulescu-Andrasi A.; Chambers K. E.; Stoian S. A.; Bominaar E. L.; Achim C.; Dunbar K. R. Properties of Prussian Blue Materials Manifested in Molecular Complexes: Observation of Cyanide Linkage Isomerism and Spin-Crossover Behavior in Pentanuclear Cyanide Clusters. J. Am. Chem. Soc. 2007, 129, 6104–6116. 10.1021/ja066273x. 17455931

[ref22] Sayin E.; Kürkçüoǧlu G. S.; Yeşilel O. Z.; Taş M.; Öztürk M.; Yerli Y. Synthesis, Crystal Structure and Magnetic Property of One-Dimensional Heterometallic Cyanide-Bridged Complex: {[Cu(HmpH)2Cu(Hmp)(HmpH)Co(μ-CN)4(CN)2]·2H_2_O}n. Polyhedron 2016, 115, 67–75. 10.1016/j.poly.2016.04.035.

[ref23] Babeshkin K. A.; Gavrikov A. V.; Petrosyants S. P.; Ilyukhin A. B.; Belova E. V.; Efimov N. N. Unexpected Supremacy of Non-Dysprosium Single-Ion Magnets within a Series of Isomorphic Lanthanide Cyanocobaltate(III) Complexes. Eur. J. Inorg. Chem. 2020, 2020, 4380–4390. 10.1002/ejic.202000798.

[ref24] Qin Y. L.; Yao C. Z.; Yang B. W.; Qin J. F.; Gong Q. J. Synthesis, Crystal Structure and Magnetic Properties of a Two-Dimensional Cyanide-Based Heterometallic Coordination Polymer with Cationic Piperazinium Ligands: Poly[Aquatetra-M2-Cyanido-K8 C:N-Dicyanido-K2 C-(Piperazinium-KN 4)Cobalt(III)Copper(II)]. Acta Crystallogr. C. Struct. Chem. 2016, 72, 21–27. 10.1107/S2053229615021361. 26742823

[ref25] Korkmaz S. A.; Karadag A.; Yerli Y.; Soylu M. S. Synthesis and Characterization of New Heterometallic Cyanido Complexes Based on [Co(CN)6]3- Building Blocks: Crystal Structure of [Cu2(N-Bishydeten)2Co(CN)6]·3H_2_O Having a Strong Antiferromagnetic Exchange. New J. Chem. 2014, 38, 5402–5410. 10.1039/C4NJ00737A.

[ref26] Li S. T.; Zhao C. C.; Cui A. L.; Kou H. Z. Cyanide-Bridged One-Dimensional Bimetallic Complexes Based on a Tridentate Copper(II) Building Block: Synthesis, Crystal Structures and Magnetic Properties. Trans. Met. Chem. 2014, 39, 387–392. 10.1007/s11243-014-9812-2.

[ref27] Zhan S. Z.; Sun D. S.; Wang J. G.; Zhou J. Y.; Liang A. Q.; Su J. Y. Synthesis, Crystal Structures and Magnetic Properties of a Series of New Cyano-Bridged Complexes Derived from Templates [Ni(CN)4]2- and [Co(III)(CN)6]3-. J. Coord. Chem. 2008, 61, 550–562. 10.1080/00958970701364826.

[ref28] Wong-Ng W.; Culp J. T.; Siderius D. W.; Chen Y. S. Synthesis and Synchrotron X-Ray Characterization of Two 2D Hoffman Related Compounds [Ni(p-Xylylenediamine)nNi(CN)4] and [Ni(p-Tetrafluoroxylylenediamine)nNi(CN)4]. Solid State Sci. 2018, 81, 12–18. 10.1016/j.solidstatesciences.2018.04.009. 32116468 PMC7047631

[ref29] Karaaǧaç D.; Kürkçüoǧlu G. S.; Yeşilel O. Z.; Hökelek T. Two Dimensional Cyano-Bridged Hetero-Metallic Coordination Polymers Containing Metal•••π Interactions. Spectrochim. Acta A Mol. Biomol. Spectrosc. 2014, 121, 196–204. 10.1016/j.saa.2013.10.033. 24239763

[ref30] Solanki D.; Hogarth G. Synthesis and Molecular Structure of [Cu(NH3)4][Ni(CN)4]: A Missing Piece in the [Cu(NH3)n][Ni(CN)4] Story. J. Mol. Struct. 2015, 1099, 388–392. 10.1016/j.molstruc.2015.06.038.

[ref31] Kartal Z. Synthesis, Spectroscopic, Thermal and Structural Properties of [M(3-Aminopyridine)2Ni(μ-CN)2(CN)2]n (M(II) = Co and Cu) Heteropolynuclear Cyano-Bridged Complexes. Spectrochim. Acta. A Mol. Biomol. Spectrosc. 2016, 152, 577–583. 10.1016/j.saa.2014.12.117. 25619856

[ref32] Senocak A.; Karadag A.; Soylu M. S.; Andac O. Two Novel Cyanido-Bridged Polymeric Complexes with Suspension Bridge Type Connections and a Series of Related Complex Salts: Crystallographic and Thermal Characterizations. New J. Chem. 2015, 39, 3675–3686. 10.1039/C5NJ00071H.

[ref33] Černák J.; Kuchár J.; Stolárová M.; Kajňaková M.; Vavra M.; Potočňák I.; Falvello L. R.; Tomás M. Preparation, Spectroscopic and Magnetic Characterization of Cu(Cyclam)M(CN)4 Complexes Exhibiting One-Dimensional Crystal Structures (Cyclam 5 1,4,8,11-Tetraazacyclotetradecane, M = Ni, Pd, Pt). Trans. Met. Chem. 2010, 35, 737–744. 10.1007/s11243-010-9387-5.

[ref34] Ghoshal D.; Ghosh A. K.; Maji T. K.; Ribas J.; Mostafa G.; Zangrando E.; Ray Chaudhuri N. Different Topologies in Heterometallic Frameworks of Copper(II) with Bridging Ligand: Syntheses, Crystal Structures, Thermal and Magnetic Properties. Inorg. Chim. Acta 2006, 359, 593–602. 10.1016/j.ica.2005.09.056.

[ref35] Mukherjeee P. S.; Kumar Maji T.; Mallah T.; Zangrando E.; Randaccio L.; Chaudhuri N. R. A novel bimetallic alternating chain: synthesis, crystal structure and magnetic study. Inorg. Chim. Acta 2001, 315, 249–253. 10.1016/S0020-1693(01)00354-1.

[ref36] Xia J.; Li T. T.; Zhao X. Q.; Wei J. F. Cyano-Bridged Heterometallic Complexes Directed by Binuclear Copper Units. J. Coord. Chem. 2013, 66, 539–550. 10.1080/00958972.2012.763118.

[ref37] Zhang G. F.; Zhou Q. P.; Dou Y. L.; Wang Y.; Wu L. P. Syntheses, Crystal Structures and Magnetic Properties of Three Low-Dimensional Materials Constructed from [Cu2(Dmpzpo)2]2+ and [M(CN)2/4]-/2-(M = Ag or Ni) Precursors (Hdmpzpo = 1,3-Bis(3,5-Dimethylpyrazol-l-Yl)Propan-2-Ol). Z. Anorg. Allg. Chem. 2007, 633, 2104–2108. 10.1002/zaac.200700248.

[ref38] Černák J.; Skorsepa J.; Abboud K.A.; Meisel M.W.; Orendac M.; Orendacova A.; Feher A. Preparation, crystal structure and magnetic properties of Cu(en)2Pd(CN)4. Inorganica Chimica Acta 2001, 326, 3–8. 10.1016/S0020-1693(01)00574-6.

[ref39] Karadag A.; Korkmaz A.; Andac O.; Yerli Y.; Topcu Y. Cyano-complexes and salts with tetracyanonickellate II and N,N-bis(2-hydroxyethyl)-ethylenediamine: Synthesis, IR spectra, magnetic properties, thermal analyses, and crystal structures. J. Coord. Chem. 2012, 65, 1685–1699. 10.1080/00958972.2012.678337.

[ref40] Kurihara H.; Nishikiori S.; Iwamoto T. Monodentate Ligation of Tetracyanonickelate(II): (2-Aminoethanol)[N-(2-aminoethyl)-1,2-ethanediamine][tetracyanonickelato(II)]copper(II)-Water (1/2). Acta Crystallogr. 1997, 53, 1409–1411. 10.1107/S010827019700632X.

[ref41] Paraschiv C.; Andruh M.; Ferlay S.; Hosseini M. W.; Kyritsakas N.; Planeix J. M.; Stanica N. Alkoxo-bridged copper(II) complexes as nodes in designing solid-state architectures. The interplay of coordinate and d10-d10 metal-metal interactions in sustaining supramolecular solid-state architectures. Dalton Trans. 2005, 1195–1202. 10.1039/B500231A. 15782254

[ref42] Çetinkaya F.; Kürkçüoglu G. S.; Yeşilel O. Z.; Hökelek T.; Dal H. One-Dimensional Cyano-Bridged Heterometallic (Cu/Ni and Cu/Pd) Complexes with 1-Ethylimidazole. Polyhedron 2012, 47, 126–133. 10.1016/j.poly.2012.08.041.

[ref43] Wang J. H.; Li Z. Y.; Yamashita M.; Bu X. H. Recent Progress on Cyano-Bridged Transition-Metal-Based Single-Molecule Magnets and Single-Chain Magnets. Coord. Chem. Rev. 2021, 428, 213617 10.1016/j.ccr.2020.213617.

[ref44] Seppälä P.; Colacio E.; Mota A. J.; Sillanpää R. Structural Diversity Due to Amino Alcohol Ligands Leading to Rare M4-Hydroxo-Bridged Tetranuclear and “Bicapped Cubane” Cores in Copper(II) Complexes: A Theoretical and Experimental Magnetostructural Study. Inorg. Chem. 2013, 52, 11096–11109. 10.1021/ic401325b. 24041252

[ref45] Karabach Y. Y.; Guedes Da Silva M. F. C.; Kopylovich M. N.; Gil-Hernández B.; Sanchiz J.; Kirillov A. M.; Pombeiro A. J. L. Self-Assembled 3D Heterometallic CuII/FeII Coordination Polymers with Octahedral Net Skeletons: Structural Features, Molecular Magnetism, Thermal and Oxidation Catalytic Properties. Inorg. Chem. 2010, 49, 11096–11105. 10.1021/ic101668f. 21028781

[ref46] Rigaku Oxford Diffraction, CrysAlisPro Software System, UK, 2022.

[ref47] Bruker. APEX4 and SAINT V8.40B; Bruker AXS Inc.: Madison, WI, 2022.

[ref48] Sheldrick G. M. SHELXT - Integrated Space-Group and Crystal-Structure Determination. Acta Crystallogr. 2015, A71, 3–8. 10.1107/S2053273314026370 PMC4283466 25537383

[ref49] Sheldrick G. M. Crystal Structure Refinement with SHELXL. Acta Crystallogr. 2015, C71, 3–8. 10.1107/S2053229614024218. PMC4294323 25567568

[ref50] Dolomanov O. V.; Bourhis L. J.; Gildea R. J.; Howard J. A. K.; Puschmann H. OLEX2: A Complete Structure Solution, Refinement and Analysis Program. J. Appl. Crystallogr. 2009, 42, 339–341. 10.1107/S0021889808042726.

[ref51] Neese F. Software Update: The ORCA Program System—Version 5.0. Wiley Interdisciplinary Reviews: Computational Molecular Science 2022, 12, e1606 10.1002/wcms.1606.

[ref52] Neese F. The ORCA Program System. Wiley Interdisciplinary Reviews: Computational Molecular Science 2012, 2, 73–78. 10.1002/wcms.81.

[ref53] Stephens P. J.; Devlin F. J.; Chabalowski C. F.; Frisch M. J. Ab Initio Calculation of Vibrational Absorption and Circular Dichroism Spectra Using Density Functional Force Fields. J. Phys. Chem. 1994, 98, 11623–11627. 10.1021/j100096a001.

[ref54] Hertwig R. H.; Koch W. On the parameterization of the local correlation functional. What is Becke-3-LYP?. Chem. Phys. Lett. 1997, 268, 345–351. 10.1016/S0009-2614(97)00207-8.

[ref55] Zheng J.; Xu X.; Truhlar D. G. Minimally Augmented Karlsruhe Basis Sets. Theor. Chem. Acc. 2011, 128, 295–305. 10.1007/s00214-010-0846-z.

[ref56] Weigend F.; Ahlrichs R. Balanced Basis Sets of Split Valence, Triple Zeta Valence and Quadruple Zeta Valence Quality for H to Rn: Design and Assessment of Accuracy. Phys. Chem. Chem. Phys. 2005, 7, 3297–3305. 10.1039/b508541a. 16240044

[ref57] Stoychev G. L.; Auer A. A.; Neese F. Automatic Generation of Auxiliary Basis Sets. J. Chem. Theory Comput. 2017, 13, 554–562. 10.1021/acs.jctc.6b01041. 28005364

[ref58] Barone V.; Cossi M. Quantum Calculation of Molecular Energies and Energy Gradients in Solution by a Conductor Solvent Model. J. Phys. Chem. A 1998, 102, 1995–2001. 10.1021/jp9716997.

[ref59] Caldeweyher E.; Ehlert S.; Hansen A.; Neugebauer H.; Spicher S.; Bannwarth C.; Grimme S. A Generally Applicable Atomic-Charge Dependent London Dispersion Correction. J. Chem. Phys. 2019, 150, 154122 10.1063/1.5090222. 31005066

[ref60] Caldeweyher E.; Bannwarth C.; Grimme S. Extension of the D3 Dispersion Coefficient Model. J. Chem. Phys. 2017, 147, 034112 10.1063/1.4993215. 28734285

[ref61] Avogadro: an open-source molecular builder and visualization tool, Ver 1.2. http://avogadro.cc/ (accessed 2023-07-23).

[ref62] Hanwell M. D.; Curtis D. E.; Lonie D. C.; Vandermeersch T.; Zurek E.; Hutchison G. R. SOFTWARE Open Access Avogadro: An Advanced Semantic Chemical Editor, Visualization, and Analysis Platform. J. Cheminform. 2012, 4, 17 10.1186/1758-2946-4-17. 22889332 PMC3542060

[ref63] Yamaguchi K.; Jensen F.; Dorigo A.; Houk K. N. A Spin Correction Procedure for Unrestricted Hartree-Fock and Møller-Plesset Wavefunctions for Singlet Diradicals and Polyradicals. Chem. Phys. Lett. 1988, 149, 537–542. 10.1016/0009-2614(88)80378-6.

[ref64] Johnson E. R.; Keinan S.; Mori-Sánchez P.; Contreras-García J.; Cohen A. J.; Yang W. Revealing Noncovalent Interactions. J. Am. Chem. Soc. 2010, 132, 6498–6506. 10.1021/ja100936w. 20394428 PMC2864795

[ref65] Lu T.; Chen F. Multiwfn: A Multifunctional Wavefunction Analyzer. J. Comput. Chem. 2012, 33, 580–592. 10.1002/jcc.22885. 22162017

[ref66] Cernak J. C.; Lipkowski J. Two Polymorphs of Cu(Tn)Ni(CN)4 Containing Tetracyanonickellate Anions with Triple Bridging Function: Preparation and Crystallographic Characterization. Monatsh. Chem. 1999, 130, 1195–1206. 10.1007/PL00010181.

[ref67] Vafazadeh R.; Dehghani-Firouzabadi A.; Willis A. C. Synthesis of Hetero- and Homo-Multinuclear Complexes with a Tetracyanonickelate Anion: Structural Characterization [Cu(Bcen)Ni(CN)4]2. Acta Chim. Slov. 2017, 64, 686–691. 10.17344/acsi.2017.3577. 28862307

[ref68] Curtis N. F.; Flood K.; Robinson W. T. N-Rac-(5,7,7,12,12,14-Hexamethyl-1,4,8,11-Tetraazacyclotetradeca-4,14(1)-Diene)Copper(II): Structures of the Perchlorate Salt and a Trinuclear Tetracyanonickelato-Bridged Compound. Polyhedron 2006, 25, 1579–1584. 10.1016/j.poly.2005.10.025.

[ref69] Gu J.-Z.; Kou H.-Z.; Jiang L.; Lu T.-B.; Tan M.-Y. Synthesis, Structures and Magnetic Properties of Pentanuclear and Trinuclear Heterometallic Complexes with Bended and Linear Cyano-Bridges (Tren = tris(2-Aminoethyl)Amine). Inorg. Chim. Acta 2006, 359, 2015–2022. 10.1016/j.ica.2005.12.078.

[ref70] Addison A. W.; Rao T. N.; Reedijk J.; van Rijn J.; Verschoor G. C. Synthesis, Structure, and Spectroscopic Properties of Copper(II) Compounds Containing Nitrogen-Sulphur Donor Ligands; the Crystal and Molecular Structure of Aqua[1,7-bis(N-methylbenzimidazol-2′-yl)-2,6-dithiaheptane]copper(II) Perchlorate. J. Chem. Soc., Dalton Trans. 1984, 1349–1356. 10.1039/DT9840001349.

[ref71] Yang L.; Powell D. R.; Houser R. P. Structural Variation in Copper(i) Complexes with Pyridylmethylamide Ligands: Structural Analysis with a New Four-Coordinate Geometry Index, Τ4. Dalton Trans. 2007, 9, 955–964. 10.1039/B617136B. 17308676

[ref72] O’Keeffe M.; Peškov M. A.; Ramsden S.; Yaghi O. M. The Reticular Chemistry Structure Resource (RCSR) Database of, and Symbols for Crystal Nets. Acc. Chem. Res. 2008, 41, 1782–1789. 10.1021/ar800124u. 18834152

[ref73] Boulsourani Z.; Tangoulis V.; Raptopoulou C. P.; Psycharis V.; Dendrinou-Samara C. Ferromagnetic and Antiferromagnetic Copper(Ii) Complexes: Counterplay between Zero-Field Effects of the Quartet Ground State and Intermolecular Interactions. Dalton Trans. 2011, 40, 7946–7956. 10.1039/c1dt10254k. 21725554

[ref74] Farrugia L. J.; Middlemiss D. S.; Sillanpää R.; Seppilälä P. A Combined Experimental and Theoretical Charge Density Study of the Chemical Bonding and Magnetism in 3-Amino-Propanolato Cu(II) Complexes Containing Weakly Coordinated Anions. J. Phys. Chem. A 2008, 112, 9050–9067. 10.1021/jp804865j. 18759408

[ref75] Seppälä P.; Colacio E.; Mota A. J.; Sillanpää R. Dinuclear Alkoxo-Bridged Copper(II) Coordination Polymers: Syntheses, Structural and Magnetic Properties. Inorg. Chim. Acta 2010, 363, 755–762. 10.1016/j.ica.2009.11.035.

[ref76] Rodríguez-Fortea A.; Alemany P.; Alvarez S.; Ruiz E. Exchange Coupling in Carboxylato-Bridged Dinuclear Copper(II) Compounds: A Density Functional Study. Chem.—Eur. J. 2001, 7, 627–637. 10.1002/1521-3765(20010202)7:3<627::AID-CHEM627>3.0.CO;2-I. 11261660

[ref77] Kahn O. Molecular Magnetism; VCH: New York, 1993.

[ref78] Seppälä P.; Sillanpää R.; Lehtonen A. Structural Diversity of Copper(II) Amino Alcoholate Complexes. Coord. Chem. Rev. 2017, 347, 98–114. 10.1016/j.ccr.2017.06.022.

[ref79] Hatfield W. E. New Magnetic and Structural Results for Uniformly Spaced, Alternatingly Spaced, and Ladder-like Copper (II) Linear Chain Compounds. J. Appl. Phys. 1981, 52, 1985–1990. 10.1063/1.329592.

[ref80] Doyle R. P.; Julve M.; Lloret F.; Nieuwenhuyzen M.; Kruger P. E. Hydrogen-Bond Tuning of Ferromagnetic Interactions: Synthesis, Structure and Magnetic Properties of Polynuclear Copper(II) Complexes Incorporating p-Block Oxo-Anions. Dalton Trans. 2006, 17, 2081–2088. 10.1039/b515889c. 16625252

[ref81] MacLaren J. K.; Sanchiz J.; Gili P.; Janiak C. Hydrophobic-Exterior Layer Structures and Magnetic Properties of Trinuclear Copper Complexes with Chiral Amino Alcoholate Ligands. New J. Chem. 2012, 36, 1596–1609. 10.1039/c2nj40063d.

[ref82] Noodleman L. Valence Bond Description of Antiferromagnetic Coupling in Transition Metal Dimers. J. Chem. Phys. 1981, 74, 5737–5743. 10.1063/1.440939.

[ref83] Noodleman L.; Davidson E. R. Ligand Spin Polarization and Antiferromagnetic Coupling in Transition Metal Dimers. Chem. Phys. 1986, 109, 131–143. 10.1016/0301-0104(86)80192-6.

[ref84] Neese F. Definition of Corresponding Orbitals and the Diradical Character in Broken Symmetry DFT Calculations on Spin Coupled Systems. J. Phys. Chem. Solids 2004, 65, 781–785. 10.1016/j.jpcs.2003.11.015.

[ref85] Ruiz E. Exchange Coupling Constants Using Density Functional Theory: Long-Range Corrected Functionals. J. Comput. Chem. 2011, 32, 1998–2004. 10.1002/jcc.21788. 21469163

[ref86] Nesterova O. V.; Vassilyeva O. Yu.; Skelton B. W.; Bieńko A.; Pombeiro A. J. L.; Nesterov D. S. A novel *o*-vanillin Fe^III^ complex catalytically active in C–H oxidation: exploring the magnetic exchange interactions and spectroscopic properties with different DFT functionals. Dalton Trans. 2021, 50, 14782–14796. 10.1039/D1DT02366G. 34595485

[ref87] Shil S.; Herrmann C. Performance of Range-separated Hybrid Exchange–Correlation Functionals for the Calculation of Magnetic Exchange Coupling Constants of Organic Diradicals. J. Comput. Chem. 2018, 39, 780–787. 10.1002/jcc.25153. 29280154

[ref88] Deeth R. J. D-Orbital Energy Levels in Planar [M^II^F_4_]^2–^, [M^II^ (NH_3_)_4_]^2+^ and [M^II^(CN)_4_]^2–^ Complexes: The Nature of M–L π Bonding and the Implications for Ligand Field Theory. Dalton Trans. 2020, 49, 9641–9650. 10.1039/D0DT02022B. 32618313

[ref89] Oppenheim J. J.; McNicholas B. J.; Miller J.; Gray H. B. Electronic Structure of Tetracyanonickelate(II). Inorg. Chem. 2019, 58, 15202–15206. 10.1021/acs.inorgchem.9b02135. 31697485

[ref90] Hay P. J.; Thibeault J. C.; Hoffmann R. Orbital Interactions in Metal Dimer Complexes. J. Am. Chem. Soc. 1975, 97, 4884–4899. 10.1021/ja00850a018.

[ref91] Zhao Y.; Truhlar D. G. A new local density functional for main-group thermochemistry, transition metal bonding, thermochemical kinetics, and noncovalent interactions. J. Chem. Phys. 2006, 125, 194101 10.1063/1.2370993. 17129083

[ref92] Furness J. W.; Kaplan A. D.; Ning J.; Perdew J. P.; Sun J. Accurate and Numerically Efficient r^2^SCAN Meta-Generalized Gradient Approximation. J. Phys. Chem. Lett. 2020, 11, 8208–8215. 10.1021/acs.jpclett.0c02405. 32876454

[ref93] Tao J.; Perdew J. P.; Staroverov V. N.; Scuseria G. E. Climbing the Density Functional Ladder: Nonempirical Meta-Generalized Gradient Approximation Designed for Molecules and Solids. Phys. Rev. Lett. 2003, 91, 146401 10.1103/PhysRevLett.91.146401. 14611541

[ref94] Adamo C.; Barone V. Toward Reliable Density Functional Methods Without Adjustable Parameters: The PBE0Model. J. Chem. Phys. 1999, 110, 6158–6170. 10.1063/1.478522.

[ref95] Pantazis D. A. Assessment of Double-Hybrid Density Functional Theory for Magnetic Exchange Coupling in Manganese Complexes. Inorganics 2019, 7, 57 10.3390/inorganics7050057.

[ref96] Bencini A.; Totti F.; Daul C. A.; Doclo K.; Fantucci P.; Barone V. Density Functional Calculations of Magnetic Exchange Interactions in Polynuclear Transition Metal Complexes. Inorg. Chem. 1997, 36, 5022–5030. 10.1021/ic961448x.

[ref97] Noodleman L.; Davidson E. R. Ligand Spin Polarization and Antiferromagnetic Coupling in Transition Metal Dimers. Chem. Phys. 1986, 109, 131–143. 10.1016/0301-0104(86)80192-6.

[ref98] Ruiz E.; Cano J.; Alvarez S.; Alemany P. J. Broken symmetry approach to calculation of exchange coupling constants for homobinuclear and heterobinuclear transition metal complexes. J. Comput. Chem. 1999, 20, 1391–1400. 10.1002/(SICI)1096-987X(199910)20:13<1391::AID-JCC6>3.0.CO;2-J.

[ref99] Allen F. H.; Bruno I. J. Bond Lengths in Organic and Metal-Organic Compounds Revisited: *X* —H Bond Lengths from Neutron Diffraction Data. Acta Crystallogr. B 2010, 66, 380–386. 10.1107/S0108768110012048. 20484809

[ref100] Weller M. T.; Henry P. F.; Ting V. P.; Wilson C. C. Crystallography of Hydrogen-Containing Compounds: Realizing the Potential of Neutron Powder Diffraction. Chem. Commun. 2009, 21, 2973–2989. 10.1039/b821336d. 19462064

[ref101] Emamian S.; Lu T.; Kruse H.; Emamian H. Exploring Nature and Predicting Strength of Hydrogen Bonds: A Correlation Analysis Between Atoms-in-Molecules Descriptors, Binding Energies, and Energy Components of Symmetry-Adapted Perturbation Theory. J. Comput. Chem. 2019, 40, 2868–2881. 10.1002/jcc.26068. 31518004

[ref102] Nanayakkara S.; Tao Y.; Kraka E. Comment on “Exploring Nature and Predicting Strength of Hydrogen Bonds: A Correlation Analysis between atoms-in-molecules Descriptors, Binding Energies, and Energy Components of symmetry-adapted Perturbation Theory. J. Comput. Chem. 2021, 42, 516–521. 10.1002/jcc.26475. 33368440

[ref103] Kuznetsov M. L. Can Halogen Bond Energy Be Reliably Estimated from Electron Density Properties at Bond Critical Point? The Case of the (A)nZ—Y···X – (X, Y = F, Cl, Br) Interactions. Int. J. Quantum Chem. 2019, 119, e25869 10.1002/qua.25869.

[ref104] Buvaylo E. A.; Nesterova O. V.; Goreshnik E. A.; Vyshniakova H. V.; Petrusenko S. R.; Nesterov D. S. Supramolecular Diversity, Theoretical Investigation and Antibacterial Activity of Cu, Co and Cd Complexes Based on the Tridentate N,N,O-Schiff Base Ligand Formed In Situ. Molecules 2022, 27, 8233 10.3390/molecules27238233. 36500325 PMC9740120

[ref105] Maurice R.; Sivalingam K.; Ganyushin D.; Guihéry N.; de Graaf C.; Neese F. Theoretical Determination of the Zero-Field Splitting in Copper Acetate Monohydrate. Inorg. Chem. 2011, 50, 6229–6236. 10.1021/ic200506q. 21634387

[ref106] Gellé A.; Munzarová M. L.; Lepetit M.-B.; Illas F. The Role of Dynamical Polarization of the Ligand to Metal Charge Transfer Excitations in in *ab initio* Determination of Effective Exchange Parameters. Phys. Rev. B 2003, 68, 125103 10.1103/PhysRevB.68.125103.

