# Supplementary material for: Alkoxy-Bridged Dicopper(II) Cores Meet Tetracyanonickelate Linkers: Structural, Magnetic, and Theoretical Investigation of Cu/Ni Coordination Polymers
Source: J Phys Chem C Nanomater Interfaces. 2024 Apr 2;128(14):6053–64. doi: 10.1021/acs.jpcc.3c08112 (PMC11017569; doi:10.1021/acs.jpcc.3c08112)
Supplement: Supplementary file 1 — jp3c08112_si_001.pdf [file jp3c08112_si_001.pdf]

Supporting Information

Alkoxy-bridged Dicopper(II) Cores Meet Tetracyanonickelate Linkers: Structural, Magnetic, and Theoretical Investigation of Cu/Ni Coordination Polymers

Ines F. M. Costa,[†,§] Chris H. J. Franco,[†] Dmytro S. Nesterov,[†] Vânia André,[†] Laura C. J. Pereira,[§*] and Alexander M. Kirillov[†*]

[†]*Centro de Química Estrutural, Institute of Molecular Sciences, Departamento de Engenharia Química, Instituto Superior Técnico, Universidade de Lisboa, Av. Rovisco Pais, 1049-001, Lisbon, Portugal; kirillov@tecnico.ulisboa.pt*

[§]*Centro de Ciências e Tecnologias Nucleares, Departmento de Engenharia Ciências Nucleares, Instituto Superior Técnico, Universidade de Lisboa, Estrada Nacional 10, 2695-066 Bobadela, Portugal; lpereira@ctn.tecnico.ulisboa.pt*

Supporting Information Contains: Materials and instruments, images of compounds (Figure S1), FTIR-ATR spectra (Fig. S2), PXRD patterns (Fig. S3-S4), TGA data (Figure S5), bonding parameters (Table S1), additional figures for **1** and **2** (Fig. S1, S6-S9) and DFT calculated Cartesian coordinates (Listings S1−S4). (PDF)

Materials and Instruments. All analytical-grade reagents were purchased from commercial sources and used as received. Synthesis of **1** and **2** was performed in air at room temperature (~298 K). Elemental analyses (EA) were carried out on a Perkin-Elmer 2400 Series II analyzer by the Laboratory of Analyses of IST. FT-IR spectra were recorded on a Shimadzu IRAffinity-1S apparatus equipped with an ATR ZnSe Performance Crystal Plate accessory; the absorbance spectra were measured in the 4000–400 cm^{-1} region with a 2.0 cm^{-1} resolution using 16 co-added scans (abbreviations: vs − very strong, s − strong, m – medium, w – weak, br – broad). Thermal analyses were performed using a Setaram model SETSYS Evolution 16 thermobalance. The crystalline samples (4.7 mg) were heated under N_2 atmosphere at the rate of 5°C/min in the 30−800°C temperature range. Powder X-ray diffraction data were acquired on a D8 Advance Bruker powder diffractometer, equipped with a LYNXEYE-XE detector, utilizing Cu Kα (λ = 1.5406 Å, Ni filter) monochromatic graphite radiation (0.6º slit, 40 kV, 30 mA). To increase count statistics and peak shape profiles, data collection parameters of 2−50° (2θ), step size = 0.02°, and scan speed = 0.17° s^{-1} were employed.

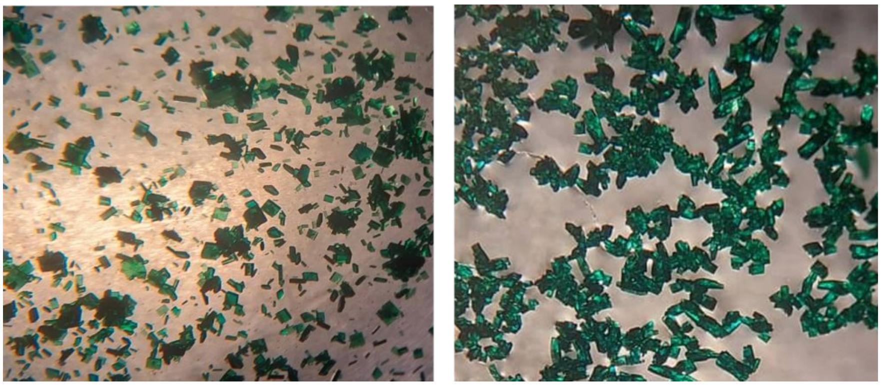

Figure S1. Images of as-synthesized microcrystalline samples of **1** (left) and **2** (right).

Table S1. Selected geometric parameters (Å) for **1** and **2**.

CP **1**			
Bond distances (Å)			
Cu1—Cu1^{i}	3.0206 (10)	Cu1—N3	2.078 (3)
Cu1—O1	2.338 (3)	Ni1—C1^{ii}	1.874 (4)
Cu1—O2^{i}	1.924 (3)	Ni1—C1	1.874 (4)
Cu1—O2	1.907 (3)	Ni1—C2	1.879 (4)
Cu1—N1	1.953 (3)	Ni1—C2^{ii}	1.879 (4)
Angles (°)			

O1—Cu1—Cu1^{i}	107.64 (7)	N1—Cu1—O1	88.03 (12)
O2^{i}—Cu1—Cu1^{i}	37.75 (8)	N1—Cu1—N3	100.92 (13)
O2—Cu1—Cu1^{i}	38.16 (8)	N3—Cu1—Cu1^{i}	121.67 (9)
O2^{i}—Cu1—O1	107.53 (12)	N3—Cu1—O1	81.62 (11)
O2—Cu1—O1	100.15 (12)	C1^{ii}—Ni1—C2	91.27 (17)
O2—Cu1—O2^{i}	75.90 (12)	C1^{ii}—Ni1—C2^{ii}	88.73 (17)
O2^{i}—Cu1—N1	98.52 (13)	C1—Ni1—C2^{ii}	91.27 (17)
O2—Cu1—N1	171.16 (13)	C1—Ni1—C2	88.73 (17)
O2^{i}—Cu1—N3	158.79 (12)	Cu1—O2—Cu1^{i}	104.09 (12)
O2—Cu1—N3	83.74 (12)		

Symmetry codes: (i) −x+1, −y+1, −z+1; (ii) −x, −y+1, −z.

CP **2**

Bond distance (Å)

Cu1—Cu1^{i}	3.0060 (5)	Cu1—N2	1.979 (2)
Cu1—O2	1.9302 (16)	Cu1—N3^{ii}	2.228 (2)
Cu1—O2^{i}	1.9433 (16)	N3—Cu1^{iii}	2.228 (2)
Cu1—N1	2.049 (2)	Cu1^{i}—N3^{iv}	2.228 (2)

Angles (°)

O2—Cu1—O2^{i}	78.20 (7)	O2^{i}—Cu1—N3^{ii}	97.89 (8)
O2—Cu1—N1	84.10 (7)	N1—Cu1—Cu1^{i}	120.84 (6)
O2^{i}—Cu1—N1	153.51 (8)	N1—Cu1—N3^{ii}	103.81 (9)
O2^{i}—Cu1—N2	95.22 (8)	N2—Cu1—Cu1^{i}	132.53 (6)
O2—Cu1—N2	163.97 (8)	N2—Cu1—N1	96.54 (8)
O2—Cu1—N3^{ii}	97.24 (8)	Cu1—O2—Cu1^{i}	101.80 (7)
O2—Cu1i—N3^{iv}	97.89 (8)		

Symmetry codes: (i) −x+1, −y+1, −z+1; (ii) x−1/2, −y+1/2, −z+1; (iii) x+1/2, −y+1/2, −z+1, (iv) x+3/2, y+1/2, z.

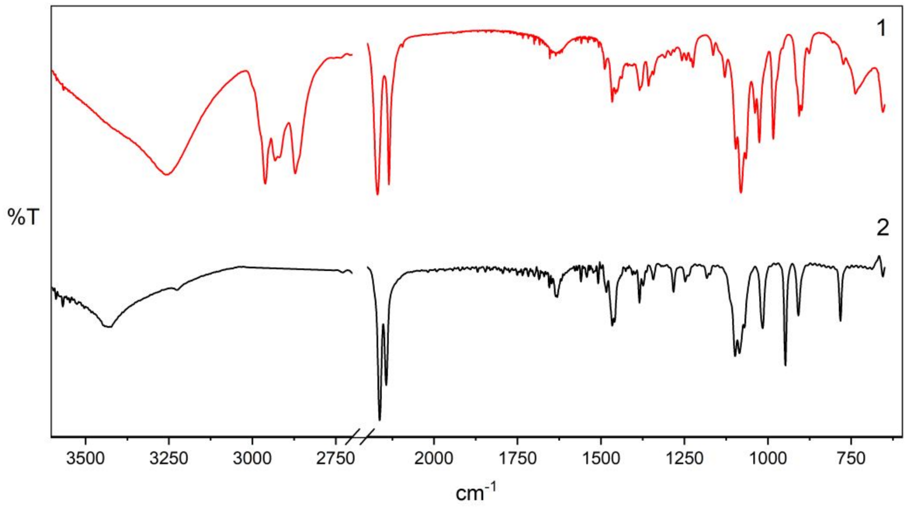

Figure S2. FTIR spectra of **1** and **2**.

FTIR Spectroscopy. The obtained compounds were also characterized by FT-IR spectroscopy (Figure S2, SI). Two strong *v*(CN) absorption bands in the range of 2000−2200 cm^{-1} with maxima at 2168 (**1**) or 2178 (**2**) cm^{-1} are typical for $[Ni(CN)_4]^{2-}$ moieties.[S1−S3] These bands appear at higher frequencies than those in $K_2[Ni(CN)_4]$ (2120 cm^{-1}),[S4] thus indicating the coordination of CN groups to copper centers. The absorption bands at 3600−3300 cm^{-1} are assigned to *v*(OH/H_2O) vibrations.[S1,S4] Additional absorption bands assigned to *v*(CH) vibrations are also observed in the 2990−2800 cm^{-1} region.[S1,S5] Furthermore, multiple bands in the 1600−800 cm^{-1} range represent the *v*(C−X) (X = C, N, O) vibrations of aminoalcoholate ligands.[S5]

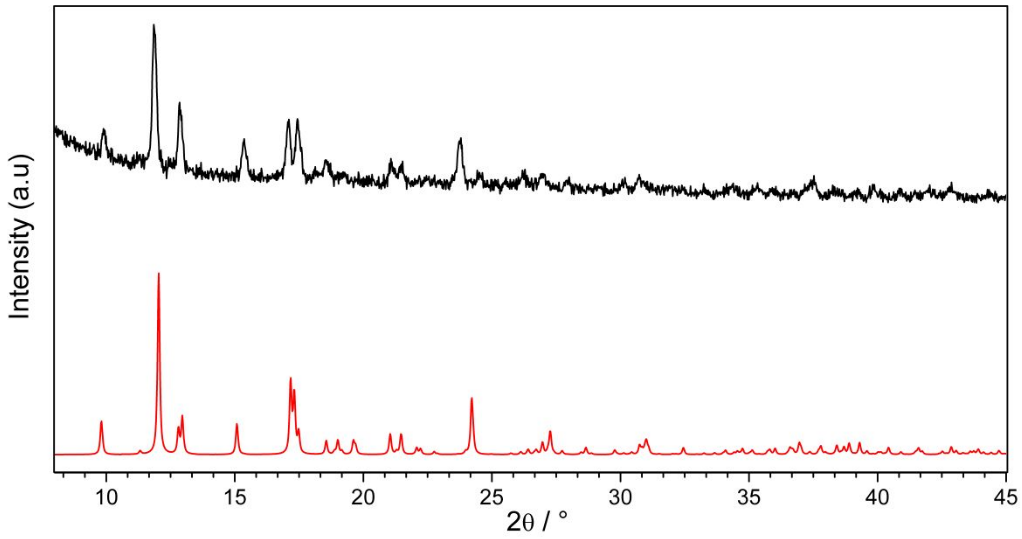

Figure S3. PXRD data for **1**: experimental (top) and calculated (down) patterns.

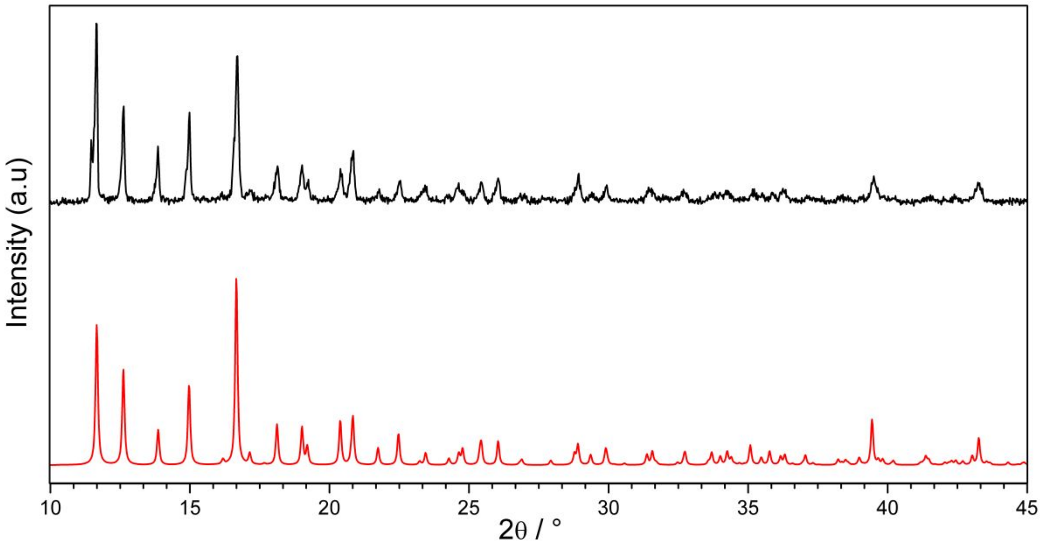

Figure S4. PXRD data for **2**: experimental (top) and calculated (down) patterns.

Thermogravimetric Analysis. To evaluate the thermal stability of **1** and **2**, TGA was run by heating the samples at a rate of 5 ºC/min from ambient temperature to 600 ºC under nitrogen atmosphere. The TGA curves are shown in Figure 3 as a percentage mass loss (%) vs. temperature (ºC). TGA of **2** reveals several thermal effects, including dehydration at 85−100°C with the release of one crystallization water molecule at 152-192°C (8.6% calcd., 8.0% observed), followed by stepwise decomposition of sample starting at 175 ºC. This process is complete at 450 ºC, resulting in the residual product corresponding to a mixture of metal oxides (2CuO+NiO; 48.6% calcd., 48.0 % observed). The TGA curve of **1** reveals a resembling behavior with thermal effects in the 160−500 ºC range. The final residue corresponds to a mixture of oxides 2CuO+NiO (38.9% calcd., 38.5% observed).

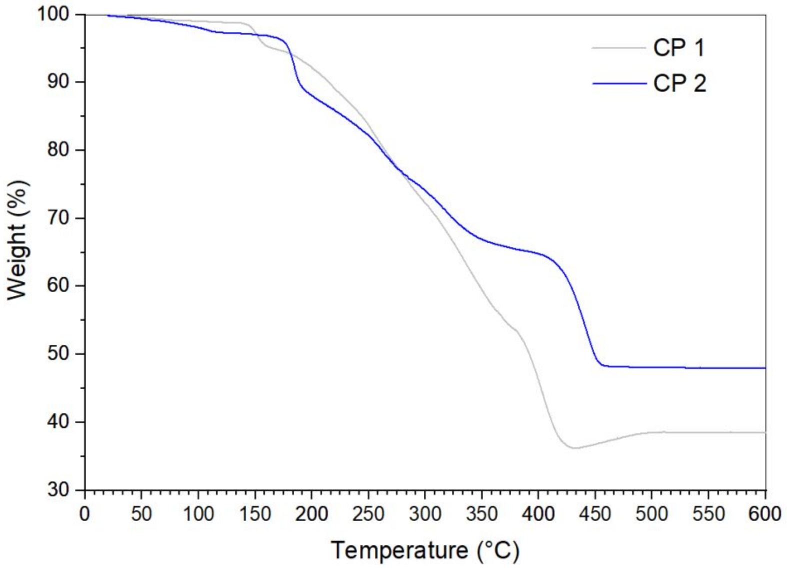

Figure S5. Thermogravimetric analysis (TGA) of **1** and **2**.

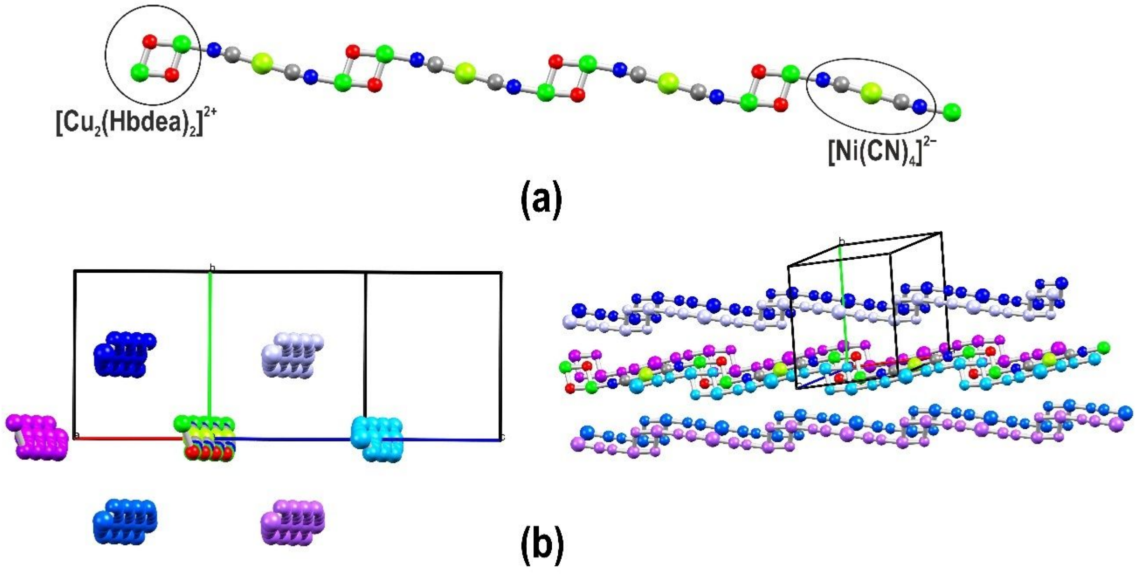

Figure S6. (a) Fragments of coordination polymeric 1D chain for **1** and (b) their topological representations for $[Cu_2(Hbdea)_2]^{2+}$ and $[Ni(CN)_4]^{2-}$ units. Two different crystallographic views for 1D chains represented by different colors.

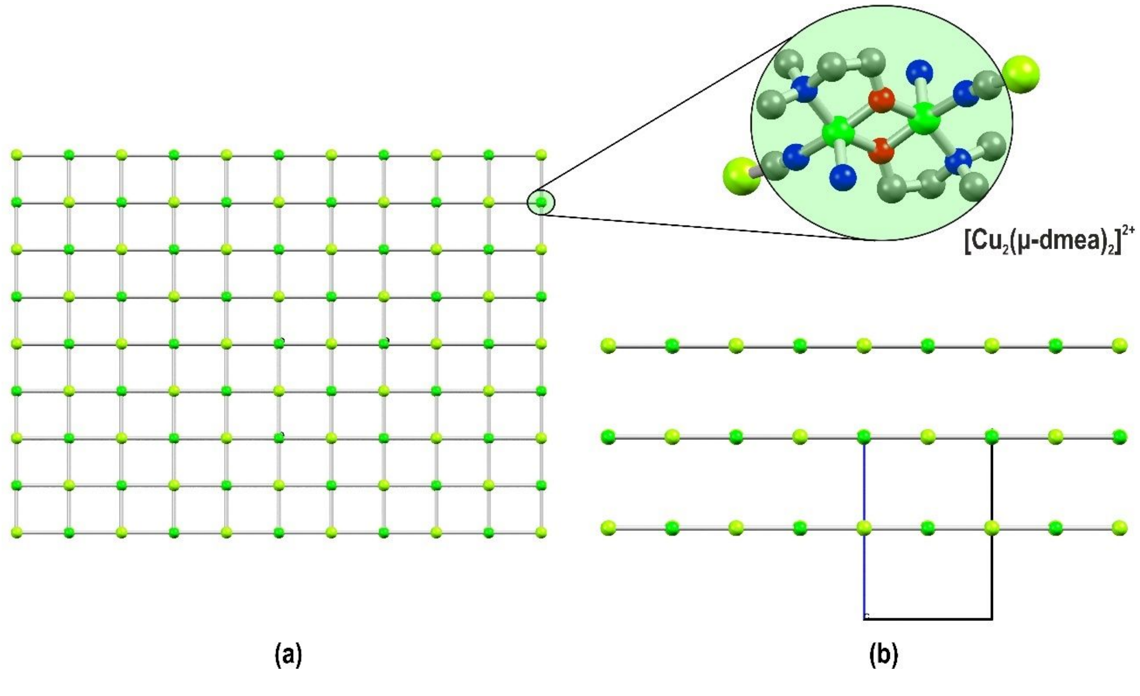

Figure S7. Topological representation for **2**. An example of simplification of $[Cu_2(\mu\text{-dmea})_2]^{2+}$ units to **sql** topology net.

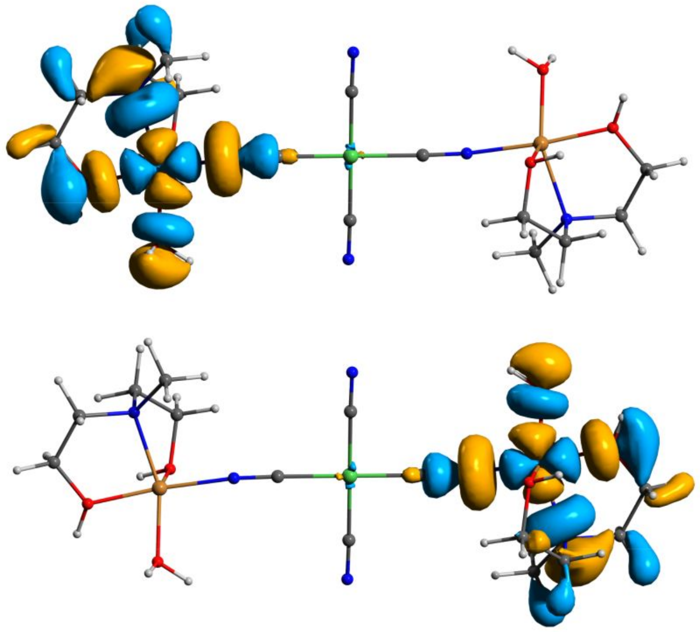

Figure S8. Isosurfaces of the unrestricted corresponding magnetic orbitals for model Cu–NC–Ni–CN–Cu fragment obtained from the B3LYP/ma-def2-TZVPP broken symmetry calculations.

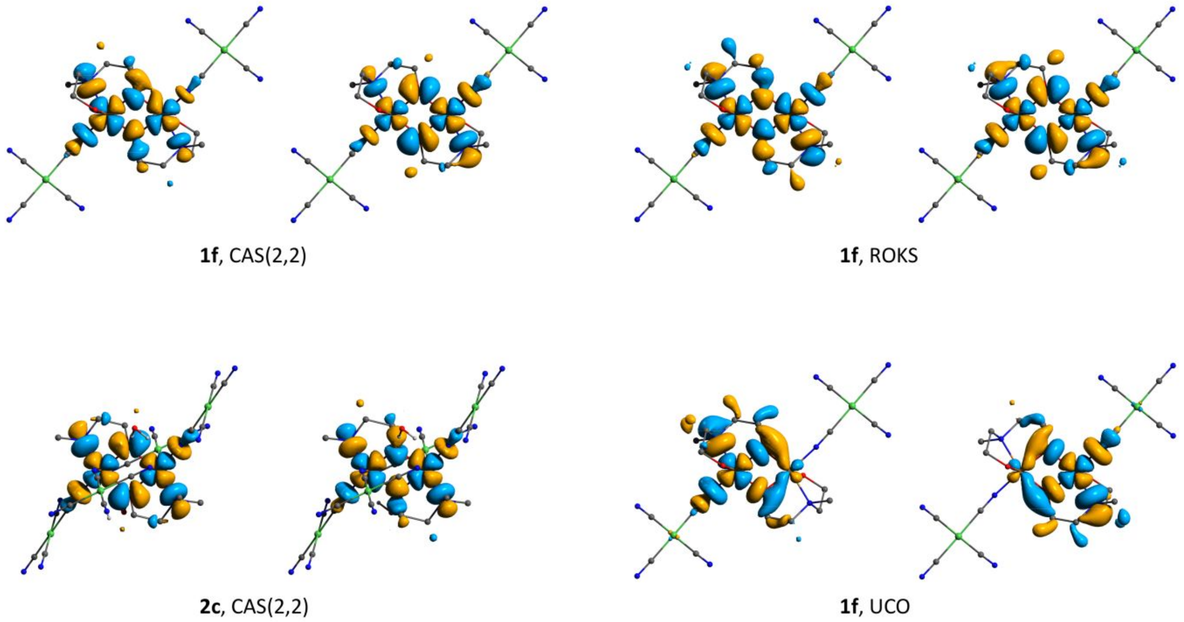

Figure S9. Top left: isosurfaces of the active space molecular orbitals (left), singly occupied molecular orbitals (top right), α and β unrestricted corresponding magnetic orbitals (bottom right) for the stated model fragments.

Listing S1. Löwdin reduced active molecular orbitals from the SA-CAS(18,10) calculation for **1f**.

```
                      166       167
                   -0.60552  -0.59840
                    1.85018   1.82736
                   --------  --------
 0 Cu dz2              0.0       5.9
 0 Cu dxz              0.0      39.5
 1 Cu dz2             16.3       5.2
 1 Cu dxz             76.5       0.6
 1 Cu dyz              4.8      43.5
 2 O  pz               0.0       0.0
 3 O  pz               0.4       0.0
 6 N  px               0.0       0.0
 7 N  px               0.0       0.0
29 H  s                0.0       0.0
31 H  s                0.0       0.0

                      168       169       170       171       172       173
                   -0.59047  -0.58701  -0.57324  -0.52377  -0.56491  -0.56273
                    1.82486   1.82270   1.79997   1.78029   1.77722   1.77497
                   --------  --------  --------  --------  --------  --------
 0 Cu dz2             13.3       7.6      49.2       0.1       9.6       0.1
 0 Cu dxz              3.2      35.3      15.6       0.1       2.0       0.0
 0 Cu dyz             79.9       6.3       6.5       0.2       0.1       0.0
 0 Cu dx2y2            0.0       0.2       4.5      37.2       8.2       0.0
 0 Cu dxy              1.0       0.0      21.2       9.2      29.6       0.0
 1 Cu dz2              0.4       4.9       0.2       0.1      29.2      11.6
 1 Cu dxz              0.4       0.2       0.0       0.0       8.4       4.6
 1 Cu dyz              0.0      42.8       0.1       0.4       1.8       2.6
 1 Cu dx2y2            0.0       0.0       0.0      36.2       1.1      19.1
 1 Cu dxy              0.0       0.6       0.2       9.9       7.7      59.9

                      174       175
                   -0.55626  -0.49777
                    1.77275   1.76969
                   --------  --------
 0 Cu s                0.1       0.0
 0 Cu dz2             11.3       0.0
 0 Cu dx2y2            7.2      35.5
 0 Cu dxy             26.8       9.5
 1 Cu s                0.3       0.0
 1 Cu dz2             29.0       0.1
 1 Cu dxz              7.9       0.0
 1 Cu dx2y2            1.3      34.9
 1 Cu dxy              8.6      10.1
12 Ni s                0.0       0.0
12 Ni pz               0.0       0.0
13 Ni s                0.0       0.0
13 Ni pz               0.0       0.0
14 C  pz               0.0       0.0
52 C  pz               0.0       0.0
```

Listing S2. Transition energies obtained from the SA-CAS(18,10) calculation for **1f**.

```
LOWEST ROOT (ROOT 0 ,MULT 1) =  -7831.528152292 Eh -213106.715 eV

STATE   ROOT MULT  DE/a.u.     DE/eV    DE/cm**-1
   1:     0    3   0.000201     0.005     44.0
   2:     1    3   0.044893     1.222   9852.8
   3:     1    1   0.045059     1.226   9889.4
   4:     2    3   0.045167     1.229   9913.0
   5:     2    1   0.045180     1.229   9915.9
   6:     3    3   0.046692     1.271  10247.7
   7:     3    1   0.046720     1.271  10253.8
   8:     4    3   0.046874     1.276  10287.6
   9:     4    1   0.046904     1.276  10294.2
  10:     5    3   0.052622     1.432  11549.2
  11:     5    1   0.052641     1.432  11553.4
  12:     6    3   0.052652     1.433  11555.8
  13:     6    1   0.052713     1.434  11569.1
  14:     7    3   0.053590     1.458  11761.6
  15:     7    1   0.053603     1.459  11764.5
  16:     8    3   0.053762     1.463  11799.5
  17:     8    1   0.053819     1.464  11812.0
```

```
  18:    9    1   0.090186     2.454  19793.5
  19:    9    3   0.090200     2.454  19796.6
  20:   10    3   0.091884     2.500  20166.1
  21:   10    1   0.091897     2.501  20169.0
  22:   11    3   0.092104     2.506  20214.4
  23:   11    1   0.092122     2.507  20218.5
  24:   12    3   0.093864     2.554  20600.7
  25:   12    1   0.093882     2.555  20604.7
  26:   13    3   0.097837     2.662  21472.7
  27:   13    1   0.097861     2.663  21478.0
  28:   14    3   0.097922     2.665  21491.4
  29:   14    1   0.097948     2.665  21497.0
  30:   15    3   0.098800     2.688  21684.2
  31:   15    1   0.098825     2.689  21689.7
  32:   16    3   0.098918     2.692  21710.0
  33:   16    1   0.098935     2.692  21713.7
  34:   17    1   0.099657     2.712  21872.2
  35:   17    3   0.099662     2.712  21873.2
  36:   18    1   0.099789     2.715  21901.2
  37:   18    3   0.099795     2.716  21902.6
  38:   19    3   0.100509     2.735  22059.3
  39:   19    1   0.100511     2.735  22059.7
  40:   20    1   0.100823     2.744  22128.2
  41:   21    1   0.105510     2.871  23156.7
  42:   22    1   0.106524     2.899  23379.4
  43:   23    1   0.106716     2.904  23421.5
  44:   24    1   0.107488     2.925  23590.9
```

Listing S3. Configurations and their weights obtained from the CAS(18,10) calculation for **1f**.

```
---------------------------------------------
CAS-SCF STATES FOR BLOCK  1 MULT= 3 NROOTS=20
---------------------------------------------

STATE   0 MULT= 3: E=  -7831.5279517651 Eh W=  0.0250 DE= 0.000 eV      0.0 cm**-1
      0.97904  : 2222212221
      0.00558  : 2222212122
      0.00481  : 2222222121
      0.00364  : 2222222211
      0.00347  : 2222211222
STATE   1 MULT= 3: E=  -7831.4832594083 Eh W=  0.0250 DE= 1.216 eV   9808.8 cm**-1
      0.44736  : 2222222211
      0.41186  : 2222211222
      0.03844  : 2222212122
      0.03751  : 2222222121
      0.01572  : 2222221221
      0.01416  : 2222212212
      0.01401  : 2222212221
      0.00618  : 2222222112
      0.00562  : 2222221122
      0.00469  : 2222221212
      0.00369  : 2222112222
STATE   2 MULT= 3: E=  -7831.4829854536 Eh W=  0.0250 DE= 1.224 eV   9869.0 cm**-1
      0.40766  : 2222221221
      0.40166  : 2222212212
      0.09675  : 2222222121
      0.08768  : 2222212122
      0.00291  : 2222221212
STATE   3 MULT= 3: E=  -7831.4814602442 Eh W=  0.0250 DE= 1.265 eV  10203.7 cm**-1
      0.32854  : 2222222121
      0.32741  : 2222212122
      0.07403  : 2222221221
      0.06245  : 2222212212
      0.05036  : 2222122221
      0.05034  : 2222222211
      0.04611  : 2222112222
      0.04352  : 2222211222
      0.00594  : 2222212221
      0.00414  : 2222221122
      0.00327  : 2222222112
STATE   4 MULT= 3: E=  -7831.4812784955 Eh W=  0.0250 DE= 1.270 eV  10243.6 cm**-1
      0.45425  : 2222122221
      0.43042  : 2222112222
      0.03936  : 2222222121
      0.02400  : 2222212122
```

```
      0.01726  : 2222211222
      0.00957  : 2222122122
      0.00922  : 2222212212
      0.00883  : 2222222211
      0.00352  : 2222221221
STATE   5 MULT= 3: E=  -7831.4755300847 Eh W=  0.0250 DE= 1.426 eV  11505.2 cm**-1
      0.21589  : 2122212222
      0.21550  : 2221222221
      0.17748  : 2221212222
      0.17504  : 2122222221
      0.11478  : 2212222221
      0.08021  : 2212212222
      0.00720  : 1222212222
STATE   6 MULT= 3: E=  -7831.4755000189 Eh W=  0.0250 DE= 1.427 eV  11511.8 cm**-1
      0.38816  : 2212222221
      0.38357  : 2212212222
      0.09102  : 2221222221
      0.06997  : 2122212222
      0.02844  : 2122222221
      0.01973  : 2221212222
      0.00808  : 2212222122
      0.00695  : 1222212222
STATE   7 MULT= 3: E=  -7831.4745623457 Eh W=  0.0250 DE= 1.453 eV  11717.6 cm**-1
      0.29457  : 2122222221
      0.28529  : 2221212222
      0.20587  : 2221222221
      0.18957  : 2122212222
      0.00572  : 2212212222
      0.00520  : 2221222122
      0.00516  : 2122222122
      0.00449  : 2212222221
STATE   8 MULT= 3: E=  -7831.4743900130 Eh W=  0.0250 DE= 1.457 eV  11755.4 cm**-1
      0.50342  : 1222222221
      0.46529  : 1222212222
      0.01306  : 2212212222
      0.00566  : 2221212222
      0.00492  : 1222221222
      0.00450  : 1222222212
STATE   9 MULT= 3: E=  -7831.4379524149 Eh W=  0.0250 DE= 2.449 eV  19752.6 cm**-1
      0.69198  : 2222221212
      0.14997  : 2222222112
      0.14062  : 2222221122
      0.00646  : 2222222211
      0.00540  : 2222211222
STATE  10 MULT= 3: E=  -7831.4362686491 Eh W=  0.0250 DE= 2.495 eV  20122.1 cm**-1
      0.33905  : 2222221122
      0.33549  : 2222222112
      0.29308  : 2222221212
      0.00788  : 2222122122
      0.00450  : 2222122212
      0.00436  : 2222221221
      0.00404  : 2222121222
      0.00375  : 2222212212
      0.00346  : 2222212122
      0.00324  : 2222222121
STATE  11 MULT= 3: E=  -7831.4360487013 Eh W=  0.0250 DE= 2.501 eV  20170.4 cm**-1
      0.36152  : 2222122212
      0.34188  : 2222121222
      0.27328  : 2222122122
      0.00652  : 2222222112
      0.00633  : 2222221122
      0.00302  : 2222221212
      0.00283  : 2222122221
STATE  12 MULT= 3: E=  -7831.4342884773 Eh W=  0.0250 DE= 2.549 eV  20556.7 cm**-1
      0.70353  : 2222122122
      0.14343  : 2222121222
      0.14106  : 2222122212
      0.00316  : 2222122221
      0.00298  : 2222221122
      0.00263  : 2222112222
STATE  13 MULT= 3: E=  -7831.4303155093 Eh W=  0.0250 DE= 2.657 eV  21428.7 cm**-1
      0.27123  : 2221222212
      0.26246  : 2122221222
      0.21759  : 2122222212
      0.21523  : 2221221222
      0.01004  : 2212221222
      0.00596  : 2212222122
```

```
      0.00328  : 2212222212
      0.00259  : 2122222221
      0.00259  : 2221212222
STATE  14 MULT= 3: E=  -7831.4302301729 Eh W=  0.0250 DE= 2.659 eV  21447.4 cm**-1
      0.34940  : 2212222212
      0.31618  : 2212221222
      0.28485  : 2212222122
      0.01572  : 2221221222
      0.01059  : 2122222212
      0.00571  : 1222221222
      0.00386  : 2221222122
      0.00286  : 2212222221
STATE  15 MULT= 3: E=  -7831.4293520056 Eh W=  0.0250 DE= 2.683 eV  21640.1 cm**-1
      0.17443  : 2122222212
      0.16327  : 2221221222
      0.15832  : 2221222212
      0.14321  : 2221222122
      0.13935  : 2122221222
      0.13890  : 2122222122
      0.03106  : 1222221222
      0.02568  : 1222222212
      0.00820  : 2212221222
      0.00450  : 2212222122
      0.00384  : 2212222212
      0.00296  : 2122122222
STATE  16 MULT= 3: E=  -7831.4292341541 Eh W=  0.0250 DE= 2.686 eV  21666.0 cm**-1
      0.46384  : 1222222212
      0.45478  : 1222221222
      0.01870  : 2221221222
      0.01212  : 2122222212
      0.01057  : 2221222122
      0.00946  : 2122222122
      0.00669  : 2122221222
      0.00570  : 2212222212
      0.00446  : 1222212222
      0.00444  : 1222222221
      0.00349  : 2221222212
STATE  17 MULT= 3: E=  -7831.4284905562 Eh W=  0.0250 DE= 2.706 eV  21829.2 cm**-1
      0.66000  : 2212222122
      0.14973  : 2212221222
      0.14242  : 2212222212
      0.01293  : 2221222122
      0.00621  : 2122222122
      0.00453  : 1222222212
      0.00441  : 2221221222
      0.00441  : 1222222122
      0.00385  : 2221222212
      0.00276  : 2212222221
STATE  18 MULT= 3: E=  -7831.4283568974 Eh W=  0.0250 DE= 2.710 eV  21858.5 cm**-1
      0.50185  : 2221122222
      0.49084  : 2122122222
STATE  19 MULT= 3: E=  -7831.4276428452 Eh W=  0.0250 DE= 2.730 eV  22015.3 cm**-1
      0.33314  : 2221222122
      0.32717  : 2122222122
      0.08022  : 2221221222
      0.07784  : 2122222212
      0.07171  : 2122221222
      0.07140  : 2221222212
      0.02267  : 2212222122
      0.00427  : 2212221222
      0.00411  : 2212222212


---------------------------------------------
CAS-SCF STATES FOR BLOCK  2 MULT= 1 NROOTS=25
---------------------------------------------

STATE   0 MULT= 1: E=  -7831.5281522916 Eh W=  0.0200 DE= 0.000 eV      0.0 cm**-1
      0.51197  : 2222222220
      0.46746  : 2222202222
      0.00573  : 2222222121
      0.00450  : 2222212122
      0.00355  : 2222221221
      0.00338  : 2222212212
STATE   1 MULT= 1: E=  -7831.4830928755 Eh W=  0.0200 DE= 1.226 eV   9889.4 cm**-1
      0.41966  : 2222222211
      0.38963  : 2222211222
```

```
      0.06248  : 2222221221
      0.05817  : 2222212212
      0.03036  : 2222212122
      0.02846  : 2222222121
STATE   2 MULT= 1: E=  -7831.4829721193 Eh W=  0.0200 DE= 1.229 eV   9915.9 cm**-1
      0.36665  : 2222221221
      0.36527  : 2222212212
      0.09709  : 2222222121
      0.09049  : 2222212122
      0.02753  : 2222222211
      0.02353  : 2222211222
      0.00597  : 2222222220
      0.00495  : 2222202222
      0.00447  : 2222221122
      0.00367  : 2222222202
      0.00366  : 2222222112
      0.00361  : 2222220222
      0.00288  : 2222122221
STATE   3 MULT= 1: E=  -7831.4814324757 Eh W=  0.0200 DE= 1.271 eV  10253.8 cm**-1
      0.34442  : 2222222121
      0.31569  : 2222212122
      0.06321  : 2222211222
      0.05811  : 2222222211
      0.05192  : 2222212212
      0.05012  : 2222221221
      0.05011  : 2222122221
      0.04951  : 2222112222
      0.00352  : 2222221122
      0.00349  : 2222222220
      0.00342  : 2222222112
STATE   4 MULT= 1: E=  -7831.4812485756 Eh W=  0.0200 DE= 1.276 eV  10294.2 cm**-1
      0.45173  : 2222122221
      0.42859  : 2222112222
      0.04230  : 2222212122
      0.03066  : 2222222121
      0.01775  : 2222221221
      0.00965  : 2222122122
      0.00826  : 2222212212
      0.00553  : 2222222211
STATE   5 MULT= 1: E=  -7831.4755111717 Eh W=  0.0200 DE= 1.432 eV  11553.4 cm**-1
      0.22270  : 2221222221
      0.19363  : 2122212222
      0.18474  : 2212212222
      0.16578  : 2212222221
      0.11724  : 2122222221
      0.10116  : 2221212222
      0.00366  : 2212222122
STATE   6 MULT= 1: E=  -7831.4754397104 Eh W=  0.0200 DE= 1.434 eV  11569.1 cm**-1
      0.33733  : 2212222221
      0.28025  : 2212212222
      0.09684  : 2221212222
      0.09045  : 2122212222
      0.08780  : 2122222221
      0.08023  : 2221222221
      0.01372  : 1222212222
      0.00651  : 2212222122
STATE   7 MULT= 1: E=  -7831.4745492403 Eh W=  0.0200 DE= 1.459 eV  11764.5 cm**-1
      0.25785  : 2122222221
      0.24168  : 2221212222
      0.19855  : 2221222221
      0.17786  : 2122212222
      0.05335  : 1222212222
      0.04624  : 1222222221
      0.00935  : 2212212222
      0.00471  : 2221222122
      0.00469  : 2122222122
      0.00321  : 2212222221
STATE   8 MULT= 1: E=  -7831.4743329590 Eh W=  0.0200 DE= 1.464 eV  11812.0 cm**-1
      0.45677  : 1222222221
      0.41416  : 1222212222
      0.04821  : 2221212222
      0.03714  : 2122222221
      0.01452  : 2122212222
      0.01012  : 2221222221
      0.00848  : 2212212222
      0.00440  : 1222221222
      0.00402  : 1222222212
```

```
STATE   9 MULT= 1: E=  -7831.4379665280 Eh W=  0.0200 DE= 2.454 eV  19793.5 cm**-1
      0.37139  : 2222222202
      0.34745  : 2222220222
      0.13374  : 2222221122
      0.12855  : 2222222112
      0.00619  : 2222221221
      0.00568  : 2222212212
      0.00296  : 2222121222
STATE  10 MULT= 1: E=  -7831.4362555739 Eh W=  0.0200 DE= 2.501 eV  20169.0 cm**-1
      0.35692  : 2222222112
      0.33475  : 2222221122
      0.13717  : 2222220222
      0.12994  : 2222222202
      0.01148  : 2222122122
      0.00666  : 2222122212
      0.00660  : 2222121222
      0.00435  : 2222222211
      0.00361  : 2222211222
      0.00353  : 2222212122
      0.00335  : 2222222121
STATE  11 MULT= 1: E=  -7831.4360299624 Eh W=  0.0200 DE= 2.507 eV  20218.5 cm**-1
      0.36029  : 2222122212
      0.33516  : 2222121222
      0.26252  : 2222122122
      0.01949  : 2222221122
      0.00605  : 2222222112
      0.00396  : 2222022222
      0.00307  : 2222222202
      0.00265  : 2222122221
STATE  12 MULT= 1: E=  -7831.4342703794 Eh W=  0.0200 DE= 2.555 eV  20604.7 cm**-1
      0.70729  : 2222122122
      0.14188  : 2222121222
      0.13854  : 2222122212
      0.00318  : 2222122221
      0.00266  : 2222112222
STATE  13 MULT= 1: E=  -7831.4302913694 Eh W=  0.0200 DE= 2.663 eV  21478.0 cm**-1
      0.24949  : 2122221222
      0.24944  : 2221222212
      0.22927  : 2221221222
      0.22550  : 2122222212
      0.01557  : 2212222212
      0.01004  : 2212222122
      0.00569  : 2212221222
      0.00250  : 2122222221
STATE  14 MULT= 1: E=  -7831.4302045997 Eh W=  0.0200 DE= 2.665 eV  21497.0 cm**-1
      0.33707  : 2212222212
      0.32002  : 2212221222
      0.27948  : 2212222122
      0.02353  : 2221222212
      0.01562  : 2122221222
      0.00525  : 1222221222
      0.00403  : 2221222122
      0.00290  : 2212222221
STATE  15 MULT= 1: E=  -7831.4293268139 Eh W=  0.0200 DE= 2.689 eV  21689.7 cm**-1
      0.18677  : 2122222212
      0.18361  : 2221221222
      0.15189  : 2221222122
      0.14740  : 2122222122
      0.14596  : 2221222212
      0.13743  : 2122221222
      0.01391  : 1222222212
      0.01029  : 1222221222
      0.00758  : 2212222212
      0.00456  : 2212221222
STATE  16 MULT= 1: E=  -7831.4292173764 Eh W=  0.0200 DE= 2.692 eV  21713.7 cm**-1
      0.47861  : 1222222212
      0.47462  : 1222221222
      0.00942  : 2212221222
      0.00931  : 2221222212
      0.00466  : 1222222221
      0.00462  : 2122221222
      0.00456  : 1222212222
      0.00411  : 2122222212
      0.00305  : 2122222122
      0.00281  : 2221222122
STATE  17 MULT= 1: E=  -7831.4284953164 Eh W=  0.0200 DE= 2.712 eV  21872.2 cm**-1
      0.66776  : 2212222122
```

```
      0.14684  : 2212221222
      0.14359  : 2212222212
      0.00994  : 2221222122
      0.00436  : 1222222122
      0.00388  : 2221221222
      0.00384  : 2122222122
      0.00295  : 2221222212
      0.00280  : 2212222221
STATE  18 MULT= 1: E=  -7831.4283629762 Eh W=  0.0200 DE= 2.715 eV  21901.2 cm**-1
      0.49492  : 2221122222
      0.48945  : 2122122222
      0.00436  : 2222022222
      0.00261  : 2212222122
STATE  19 MULT= 1: E=  -7831.4276409786 Eh W=  0.0200 DE= 2.735 eV  22059.7 cm**-1
      0.33303  : 2221222122
      0.32812  : 2122222122
      0.07854  : 2221221222
      0.07618  : 2221222212
      0.07607  : 2122221222
      0.07531  : 2122222212
      0.01633  : 2212222122
      0.00340  : 2212222212
      0.00336  : 2212221222
STATE  20 MULT= 1: E=  -7831.4273288975 Eh W=  0.0200 DE= 2.744 eV  22128.2 cm**-1
      0.98348  : 1222122222
      0.00761  : 2212122222
STATE  21 MULT= 1: E=  -7831.4226427105 Eh W=  0.0200 DE= 2.871 eV  23156.7 cm**-1
      0.48457  : 2112222222
      0.48379  : 2211222222
      0.01335  : 2220222222
      0.00522  : 2022222222
      0.00424  : 1122222222
STATE  22 MULT= 1: E=  -7831.4216279778 Eh W=  0.0200 DE= 2.899 eV  23379.4 cm**-1
      0.48371  : 2220222222
      0.47351  : 2022222222
      0.01521  : 2211222222
      0.01499  : 1212222222
      0.00476  : 2112222222
      0.00347  : 1221222222
STATE  23 MULT= 1: E=  -7831.4214361800 Eh W=  0.0200 DE= 2.904 eV  23421.5 cm**-1
      0.94008  : 1212222222
      0.01476  : 2202222222
      0.01236  : 0222222222
      0.00969  : 2220222222
      0.00870  : 1221222222
      0.00847  : 2022222222
      0.00323  : 1122222222
STATE  24 MULT= 1: E=  -7831.4206641087 Eh W=  0.0200 DE= 2.925 eV  23590.9 cm**-1
      0.49208  : 1221222222
      0.48051  : 1122222222
      0.01401  : 1212222222
      0.00547  : 2112222222
      0.00319  : 2220222222
```

Listing S4. Configurations and their weights obtained from the SS-CAS(18,10) calculation for **1f**.

```
---------------------------------------------
CAS-SCF STATES FOR BLOCK  1 MULT= 3 NROOTS= 1
---------------------------------------------

STATE   0 MULT= 3: E=  -7831.5345647370 Eh W=  1.0000 DE= 0.000 eV      0.0 cm**-1
      1.00000  : 2222222211

---------------------------------------------
CAS-SCF STATES FOR BLOCK  1 MULT= 1 NROOTS= 1
---------------------------------------------

STATE   0 MULT= 1: E=  -7831.5348413253 Eh W=  1.0000 DE= 0.000 eV      0.0 cm**-1
      0.52607  : 2222222220
      0.47393  : 2222222202
```

Listing S5. Cartesian coordinates (Å) of the fragments optimized at the B3LYP/ma-def2-TZVP level.

1a, charge -2

```
Ni -0.42405 -4.93219 -0.15731
C -0.48861 -6.80020 -0.13135
C -0.35948 -3.06417 -0.18327
C 1.16353 -4.92899 0.85308
C -2.01163 -4.93538 -1.16771
N -0.56234 -7.93862 -0.12169
N -0.28576 -1.92576 -0.19294
N 2.12977 -4.89876 1.45908
N -2.97787 -4.96562 -1.77370
Cu 0.00000 0.00000 0.00000
O 1.92579 0.00000 0.00000
O 0.45910 1.85515 -0.03963
O -0.70208 -0.20016 2.21525
N -1.93686 0.71102 -0.23443
Cu 2.38490 1.85516 -0.03964
C 2.99499 -0.90837 -0.05320
C -0.61009 2.76353 0.01356
H -0.63020 0.31838 2.93421
C -2.11031 -0.35256 2.00816
C -2.71224 -0.10953 -1.20057
C -1.76601 2.11568 -0.72651
C -2.62484 0.72650 1.08764
O 3.08698 2.05532 -2.25489
N 4.32175 1.14414 0.19479
N 2.67065 3.78092 0.15330
C 4.15091 -0.26052 0.68687
H 2.74416 -1.72481 0.45428
H 3.29154 -1.10796 -0.98778
H -0.35926 3.57997 -0.49392
H -0.90664 2.96312 0.94814
H -2.29425 -1.24174 1.61193
H -2.58132 -0.29983 2.87644
H -2.83644 -1.01520 -0.82039
H -3.60987 0.29426 -1.31040
C -2.06784 -0.24226 -2.57093
H -1.58245 2.11102 -1.69937
H -2.59684 2.62973 -0.56969
H -2.49087 1.60981 1.51452
H -3.59746 0.60003 0.94980
H 3.01510 1.53677 -2.97385
C 4.49521 2.20772 -2.04780
C 5.09714 1.96469 1.16093
C 5.00974 1.12866 -1.12728
C 2.74438 4.91933 0.14364
H 3.96735 -0.25586 1.65973
H 4.98174 -0.77457 0.53005
C -2.67465 -1.38070 -3.38524
H -2.17957 0.60810 -3.06572
H -1.09732 -0.40279 -2.45847
H 4.67915 3.09690 -1.65157
H 4.96622 2.15499 -2.91608
H 5.22134 2.87037 0.78076
H 5.99477 1.56090 1.27077
C 4.45274 2.09742 2.53130
H 4.87577 0.24535 -1.55416
H 5.98236 1.25512 -0.98942
Ni 2.80895 6.78735 0.11768
H -3.65788 -1.26725 -3.42100
H -2.48431 -2.24090 -2.93494
C -2.12500 -1.42385 -4.79606
C 5.05955 3.23586 3.34560
H 4.56447 1.24706 3.02609
H 3.48222 2.25795 2.41883
C 2.87351 8.65536 0.09172
C 4.39653 6.79054 1.12808
C 1.22136 6.78415 -0.89272
H -2.53983 -2.16360 -5.28562
H -2.32579 -0.57880 -5.25117
H -1.15509 -1.55546 -4.76447
H 6.04278 3.12241 3.38137
H 4.86921 4.09606 2.89530
C 4.50990 3.27901 4.75642
N 2.94724 9.79378 0.08205
N 5.36276 6.82078 1.73406
N 0.25513 6.75392 -1.49871
H 4.92472 4.01876 5.24598
```

H 4.71069 2.43395 5.21153
H 3.53999 3.41061 4.72484

1b, charge -2
Cu 0.00000 0.00000 0.00000
Cu 1.84109 2.37406 0.32424
Ni 0.80573 -4.88531 -0.12477
C 1.18055 -6.71561 -0.06289
C 0.42316 -3.05634 -0.17798
C 2.15261 -4.52186 1.13822
C -0.54115 -5.24876 -1.38775
N 1.37712 -7.83909 -0.03704
N 0.22184 -1.93360 -0.19856
N 2.96707 -4.27302 1.89749
N -1.35573 -5.49759 -2.14690
O 1.84109 0.46253 0.32424
O 0.00000 1.91152 -0.00000
O -0.99607 -0.40881 2.06907
N -1.98297 0.22972 -0.57179
C 3.09039 -0.16116 0.47056
C -1.24930 2.53522 -0.14632
H -1.12571 0.39285 2.59408
C -2.27089 -0.89064 1.63101
C -2.36452 -0.73282 -1.63757
C -2.07418 1.64420 -1.05693
C -2.86699 0.05210 0.61496
O 2.83716 2.78287 -1.74482
N 3.82407 2.14434 0.89604
N 1.61926 4.30766 0.52280
C 3.91527 0.72987 1.38117
H 2.98691 -1.15049 0.92716
H 3.57557 -0.30189 -0.50345
H -1.14581 3.52455 -0.60292
H -1.73448 2.67596 0.82770
H -2.09056 -1.87798 1.20947
H -2.95391 -0.99829 2.47658
H -2.31481 -1.72870 -1.19635
H -3.41029 -0.55192 -1.91254
C -1.48587 -0.67845 -2.87684
H -1.64904 1.68733 -2.05550
H -3.12276 1.95413 -1.11086
H -3.01056 1.02205 1.08957
H -3.85030 -0.30300 0.29373
H 2.96680 1.98121 -2.26984
C 4.11198 3.26470 -1.30677
C 4.20561 3.10688 1.96182
C 4.70809 2.32196 -0.29072
C 1.41794 5.43041 0.50223
H 3.49014 0.68673 2.37974
H 4.96385 0.41994 1.43510
C -1.65547 -1.91217 -3.75805
H -1.71643 0.21045 -3.46821
H -0.43469 -0.59387 -2.58385
H 3.93165 4.25205 -0.88523
H 4.79500 3.37236 -2.15234
H 4.15589 4.10277 1.52059
H 5.25139 2.92599 2.23678
C 3.32696 3.05252 3.20109
H 4.85165 1.35201 -0.76533
H 5.69139 2.67706 0.03052
Ni 1.03536 7.25938 0.44901
H -2.71942 -2.05861 -3.97502
H -1.33080 -2.79742 -3.20493
C -0.88211 -1.79279 -5.05499
C 3.49656 4.28623 4.08230
H 3.55753 2.16361 3.79246
H 2.27579 2.96793 2.90811
C 0.66054 9.08968 0.38713
C 2.38224 7.62283 1.71201
C -0.31152 6.89593 -0.81398
H -0.99952 -2.68356 -5.67633
H -1.22181 -0.93286 -5.63854
H 0.18667 -1.65974 -4.86432
H 4.56051 4.43268 4.29926
H 3.17189 5.17149 3.52917
C 2.72320 4.16685 5.37924
N 0.46397 10.21316 0.36128

N 3.19683 7.87165 2.47114
N -1.12598 6.64708 -1.57325
H 2.84059 5.05763 6.00057
H 3.06291 3.30694 5.96280
H 1.65443 4.03379 5.18856

1c, charge +4
Ni 5.35711629100313 -5.21277222039609 0.00376773473811
C 5.11671113699008 -5.55748593387762 1.82522703895625
C 5.62397514175906 -4.83183144027994 -1.80675195018085
C 6.88356610745657 -6.31213271534838 -0.04796814211849
C 3.83065633093806 -4.11341194089564 0.05551360488755
N 4.95158073778209 -5.73831651229835 2.93946808074303
N 5.80526996411227 -4.62884316021991 -2.91465085543374
N 7.80844234395245 -6.97747989763895 -0.10860248239966
N 2.90620554222388 -3.44748638435043 0.11614981576216
Cu 6.36439206421215 -4.36075993970684 -4.77017681432852
O 7.09497730390290 -6.11187392509626 -5.09960044156992
O 6.76659343339477 -4.34631218168052 -6.63885260994230
O 8.09473533597102 -3.01740668263270 -3.96916972569957
N 5.51656555670512 -2.50857663065964 -5.17331271274844
Cu 7.49717863316520 -6.09742621801268 -6.96828623248289
C 7.32257045627284 -7.32025257840358 -4.42197724256788
C 6.53900024221647 -3.13793357474407 -7.31648580173231
H 8.74617037419326 -2.79265056316466 -4.64752380626780
C 7.34945515059473 -1.84389713402159 -3.62762069287201
C 4.22226408494750 -2.32894242642024 -4.46589027731918
C 5.33161055900210 -2.49396879146632 -6.65991569661675
C 6.46641996121742 -1.42987853007025 -4.77883647080243
O 5.76683536294435 -7.44077947484769 -7.76929332335616
N 8.34500515795500 -7.94959948137018 -6.56514038151474
N 8.05630073192848 -5.82934300146481 -8.82381219235331
C 8.52996017175327 -7.96421731834606 -5.07853734859257
H 7.53645130015993 -7.14404971738987 -3.36277267192708
H 6.44023676825297 -7.96987172170876 -4.47208093297626
H 6.32501476696055 -3.31421973120844 -8.37566473392388
H 7.42135016878798 -2.48835876076206 -7.26645947028402
H 6.76895202057225 -2.10119839875264 -2.74332214568180
H 8.02116531763495 -1.02298273946304 -3.36710341557937
H 4.44503449262870 -2.30472806598889 -3.39859313179927
H 3.81373881417377 -1.34737055240868 -4.73231081703191
C 3.19502934688353 -3.41257743945303 -4.75097997453348
H 4.45442457287713 -3.08859796552907 -6.89757439771074
H 5.16262117329861 -1.46904681684764 -7.00473600450105
H 7.10197761026849 -1.19764498412826 -5.63232072730406
H 5.91041930001390 -0.52220514062272 -4.52838671177373
H 5.11521197717233 -7.66564321482134 -7.09114970592450
C 6.51211554886424 -8.61428902150788 -8.11084235762943
C 9.63930661420069 -8.12924374331462 -7.27257276246856
C 7.39515076585445 -9.02830758353221 -6.95961657777620
C 8.23760951196750 -5.62635951680153 -9.93170732173848
H 9.40719733055723 -7.36970878052310 -4.84083162446442
H 8.69880427497896 -8.98918685761534 -4.73375251158012
C 2.05818827285088 -3.41091617949021 -3.73364679742594
H 2.77260898747620 -3.28349259414171 -5.74973636871846
H 3.67632268696893 -4.39569292083982 -4.74205252840258
H 7.09255268598572 -8.35708709228647 -8.99520477504357
H 5.84037430870246 -9.43522540553030 -8.37127426013030
H 9.41648165000392 -8.15353433704828 -8.33985999871925
H 10.04776498732224 -9.11085018474225 -7.00614431485205
C 10.66655135812993 -7.04560872042007 -6.98748309583383
H 6.75954920202293 -9.26056551612085 -6.10616304441596
H 7.95110114771363 -9.93602242484471 -7.21005313719056
Ni 8.50530690875254 -5.24606660885010 -11.74224080297822
H 1.63405898291127 -2.40336832722395 -3.66412184346363
H 2.46233274948175 -3.64333882069449 -2.74485164561177
C 0.97077728099585 -4.40064153532424 -4.09746376197926
C 11.80338244011629 -7.04727000538767 -8.00481622592392
H 11.08892029325103 -7.17479074915150 -5.98872031510955
H 10.18519292186006 -6.06253494178891 -6.99637035743531
C 8.77770849672925 -4.86398269147500 -13.55169940948247
C 10.03225866623286 -6.34488087257452 -11.79129675460703
C 6.97838079059848 -4.14724267804244 -11.69322086154215
H 0.16533862748951 -4.40196345143413 -3.35950594578971
H 0.53103109882779 -4.16244965451851 -5.06970263709413
H 1.36884158130917 -5.41765105343406 -4.15626674200362
H 12.22743767864970 -8.05485630034539 -8.07434490646693

H 11.39915967440184 -6.81488269275714 -8.99356446103683
C 12.89079344879270 -6.05754466989098 -7.64099925632261
N 8.96292861238877 -4.66068220313633 -14.65888900307442
N 10.95748666504008 -7.00985876739219 -11.85030526921672
N 6.05318132229112 -3.48219578211668 -11.63424674306780
H 13.69620513244532 -6.05626123142774 -8.37897327725870
H 13.33048818603493 -6.29583433934998 -6.66876427045805
H 12.49266656893614 -5.04057358001995 -7.58216523975701
H 4.80863302860262 -5.89808520483630 3.92541578839275
H 8.62301933571947 -7.57017007215272 -0.15914750279096
H 2.09147874164803 -2.85495266083782 0.16590545464850
H 5.23883576229202 -2.88919054952323 -11.58362594001477
H 11.77297021498963 -7.60168670308038 -11.89593300495938
H 9.12841964933198 -4.47230906797593 -15.63619982244246

1e, charge 0
Cu 6.36440497127287 -4.36077001476100 -4.77015931339180
Cu 7.49715597083772 -6.09741415742578 -6.96830983804821
Ni 5.35722401693221 -5.21283479352168 0.00379969751277
C 5.11681835878944 -5.55761616715213 1.82524865445454
C 5.62405019308688 -4.83187412986925 -1.80672094180059
C 6.88367409942851 -6.31219276794179 -0.04798090802479
C 3.83076392915180 -4.11347682495115 0.05558030046989
N 4.95169101380497 -5.73848371522251 2.93948295102957
N 5.80531952530358 -4.62887146623054 -2.91462200368149
N 7.80854360639076 -6.97754121556426 -0.10863550067970
N 2.90631394779638 -3.44755904310605 0.11624489610903
O 7.09499053159904 -6.11187561071889 -5.09961256519556
O 6.76657041530965 -4.34629855999493 -6.63883658629008
O 8.09476851384058 -3.01742741473583 -3.96916615483087
N 5.51657577856937 -2.50858627116000 -5.17325276330370
C 7.32259419591733 -7.32026303744724 -4.42201039448923
C 6.53896675383876 -3.13791113259738 -7.31645875432606
H 8.74520750058693 -2.79173753633768 -4.64818852626318
C 7.34949370512191 -1.84392198172745 -3.62758914392536
C 4.22227868407859 -2.32895545046206 -4.46580682177729
C 5.33158828539276 -2.49395966541008 -6.65985917666607
C 6.46643204892723 -1.42988982043717 -4.77878815719065
O 5.76680242422537 -7.44075675242223 -7.76929299516603
N 8.34499515893774 -7.94958790283649 -6.56519638969523
N 8.05624141621451 -5.82930270151798 -8.82383714916617
C 8.52997265962560 -7.96422451026022 -5.07859997609278
H 7.53579265035635 -7.14485322483348 -3.36289589687051
H 6.44060859195302 -7.97082806786776 -4.47264856687282
H 6.32567843568272 -3.31341828128864 -8.37555371171099
H 7.42096132015757 -2.48738232264174 -7.26589444145864
H 6.76817138364374 -2.10286400378216 -2.74434089157492
H 8.02106446343556 -1.02297435862865 -3.36663192663674
H 4.44566560064738 -2.30667874321918 -3.39874295773635
H 3.81346380374781 -1.34708350200623 -4.73148446341588
C 3.19504443561641 -3.41259318899990 -4.75089009594371
H 4.45435806133848 -3.08868377303594 -6.89740061122450
H 5.16215211659748 -1.46910557224763 -7.00521533203875
H 7.10200712757097 -1.19735634322637 -5.63232438209551
H 5.91087255348442 -0.52186085467505 -4.52805951003748
H 5.11617102149682 -7.66655851236474 -7.09048525321099
C 6.51207722878219 -8.61426218952879 -8.11087000434892
C 9.63928225517661 -8.12921872887205 -7.27265233022718
C 7.39512888714734 -9.02829435196026 -6.95967099221641
C 8.23753074089173 -5.62631003322897 -9.93173820978119
H 9.40724521115101 -7.36960998532142 -4.84100335631732
H 8.69926200213325 -8.98912340309112 -4.73327100309768
C 2.05822334629619 -3.41094581261578 -3.73353894462489
H 2.77292902184569 -3.28370350035868 -5.74995586022797
H 3.67769374676257 -4.39497005977539 -4.74126310728340
H 7.09334133066147 -8.35541249132234 -8.99417546003230
H 5.84047263018306 -9.43522566307885 -8.37175333754011
H 9.41584037575313 -8.15156716001617 -8.33970509822047
H 10.04803760226493 -9.11112406319434 -7.00696904655825
C 10.66652650569557 -7.04559099232712 -6.98756905292445
H 6.75951382424581 -9.26084689248818 -6.10616016462199
H 7.95063731292882 -9.93636577153793 -7.21038148909681
Ni 8.50519690355488 -5.24599935928454 -11.74226886739458
H 1.63307621430954 -2.40332908639539 -3.66723833756524
H 2.46078932131794 -3.63955187980885 -2.74320130526459
C 0.97080754896946 -4.40066272717543 -4.09734000986353
C 11.80333759620388 -7.04723836900458 -8.00493020271786

H 11.08858585601321 -7.17456792256043 -5.98849277579483
H 10.18381518498024 -6.06325200219910 -6.99715818282101
C 8.77761825770508 -4.86381310881478 -13.55170532472208
C 10.03214703885141 -6.34480735664316 -11.79136949339442
C 6.97826676803189 -4.14717136214714 -11.69320824904961
H 0.16467681308986 -4.40098468628040 -3.36010741456888
H 0.53179355883462 -4.16376311670476 -5.07026642328438
H 1.36842596217107 -5.41799372726637 -4.15461524701101
H 12.22843992755457 -8.05489057668370 -8.07116005653456
H 11.40074519101700 -6.81875103913355 -8.99526506310115
C 12.89075339360851 -6.05751145223114 -7.64111913551188
N 8.96285130771628 -4.66045422099806 -14.65888232952580
N 10.95737746343267 -7.00978458460055 -11.85040406321864
N 6.05306722466501 -3.48213290625227 -11.63421364167656
H 13.69689666174972 -6.05727223358843 -8.37832567768369
H 13.32967237799954 -6.29445438287507 -6.66816190505059
H 12.49309090168850 -5.04020605534053 -7.58388293582656
H 4.80983057407260 -5.89812527987869 3.92265855502423
H 9.12736665983378 -4.47256807278638 -15.63347885506649

1f, charge 0
Cu -1.19245 -0.927795 0
Cu 1.19245 0.927795 -0.00063
O 0.73334 -0.927795 0
O -0.73336 0.927775 -0.00062
O -1.89452 -1.174505 2.21055
O 1.89452 1.174495 -2.21117
N -3.12931 -0.212025 -0.21942
N 3.12929 0.212005 0.21882
N -1.4782 -2.849085 -0.23338
N 1.47819 2.849065 0.23277
C -1.55191 -3.987445 -0.24765
C 1.55192 3.987445 0.24705
Ni 1.61738 5.855545 0.26092
Ni -1.61647 -5.855595 -0.26097
C -1.70423 -7.722705 -0.23783
C -0.02889 -5.873645 0.74925
C -3.20406 -5.837545 -1.27122
N -1.79209 -8.860115 -0.22986
N 0.93735 -5.856155 1.35574
N -4.17064 -5.854865 -1.87716
C 1.80254 -1.834835 -0.07229
C -1.80256 1.834835 0.07167
H -1.73524 -0.385655 2.74684
C -3.30275 -1.322525 2.00031
C -3.90469 -1.012075 -1.20261
C -2.95847 1.202685 -0.68186
C -3.81729 -0.224345 1.10267
C 2.95846 -1.202695 0.68123
H 1.5434 -2.790195 0.39488
H 2.07036 -2.042805 -1.11571
H -1.54335 2.790115 -0.39567
H -2.07029 2.042915 1.11506
H -3.43777 -2.306445 1.55391
H -3.83961 -1.301235 2.9514
H -4.0581 -2.015735 -0.81341
H -4.87621 -0.544795 -1.38896
H -2.71447 1.166235 -1.744
H -3.89553 1.754035 -0.55588
H -3.64417 0.731695 1.59559
H -4.89673 -0.324495 0.95587
H 1.73534 0.385885 -2.74786
C 3.30275 1.322515 -2.00091
C 3.90467 1.012065 1.20199
C 3.81729 0.224325 -1.1033
H 2.71463 -1.166345 1.7434
H 3.89549 -1.754075 0.55501
H 3.43787 2.306465 -1.55462
H 3.83961 1.301155 -2.95204
H 4.05805 2.015505 0.81245
H 4.87605 0.544495 1.38847
H 3.6443 -0.731665 -1.59637
H 4.89674 0.324575 -0.95656
C 1.68107 7.723645 0.28267
C 3.2042 5.835095 1.27233
C 0.03056 5.876035 -0.75049
N 1.75481 8.861925 0.30246

N 4.16993 5.851165 1.87965
N -0.93524 5.860035 -1.35771
H -1.86792 -9.863065 -0.21643
H 1.81132 9.865995 0.3225
H -3.34613 -1.074155 -2.13486
H 3.34589 1.074195 2.1341

1g, charge -2
Ni 0.79673853678861 -4.87055403249160 0.40582771803132
C 1.21097468537123 -6.68130681633262 0.61528156249879
C 0.38053786701622 -3.05944887517215 0.20328997099145
C 2.54773720375696 -4.36976317498725 0.87964912891374
C -0.95427010794520 -5.37133487517263 -0.06799365492379
N 1.42980215402078 -7.79312637534475 0.74757396951390
N 0.16049191302088 -1.94738272452633 0.07521167447741
N 3.60387074237643 -4.03907518256672 1.15714017252767
N -2.01043002764362 -5.70199172608647 -0.34537862580492
Cu -0.00457963726871 0.00085877574747 0.00793728041055
O 1.78217179681585 0.41441979446455 -0.57955959341932
O -0.05527058807067 1.88215255340232 -0.32684195109327
O 0.00563946789904 0.00081923171526 2.34036489334470
N -2.04478383171244 0.23415855104465 0.31622289347149
Cu 1.73148082189326 2.29572357014547 -0.91434882515175
C 2.98752422817550 -0.24014596858751 -0.87926454407683
C -1.26063304070744 2.53672833630767 -0.02714695756630
H 0.09714069141933 0.88555184210488 2.72093256964164
C -1.31981318219237 -0.48068578369705 2.58643626219189
C -2.82483630360103 -0.87348875790543 -0.29417412598876
C -2.37470480403432 1.54896064641057 -0.32163546813100
C -2.32211974088258 0.30648934356016 1.77885324255283
O 1.72126172079596 2.29576310969808 -3.24677643826887
N 3.77168507909249 2.06243379115144 -1.22262443543260
N 1.56640929338728 4.24397506758748 -0.98162322144611
C 4.10160599414452 0.74762169754128 -0.58477607364800
H 3.11873510633053 -1.13135243761361 -0.25755343333615
H 3.00738849129744 -0.56699096928985 -1.92653753050280
H -1.39185253521259 3.42794273053172 -0.64884091404900
H -1.28049630555059 2.86356467151037 1.02013257196833
H -1.31341196331144 -1.53343140439570 2.30835343488333
H -1.56587605335878 -0.40787373504006 3.64829904267458
H -2.60026137613216 -1.80223937710479 0.22437068383293
H -3.89668741968512 -0.66711399029349 -0.21977340852138
H -2.42617075445193 1.38043203346662 -1.39780481410330
H -3.35156093799766 1.90134303364225 0.02474992448602
H -2.27109333678779 1.34917121075076 2.08993039188298
H -3.33804332725831 -0.04604449885210 1.98007045360030
H 1.62975846824254 1.41102872193659 -3.62733679727931
C 3.04671437291096 2.77726812147025 -3.49284780783702
C 4.55173750619629 3.17008110600569 -0.61223741748056
C 4.04902093092472 1.99009299795542 -2.68526478336953
C 1.34636334145518 5.35604121860733 -1.10970151825724
H 4.15303108355853 0.91614058014184 0.49140528013377
H 5.07847967712964 0.39524980671056 -0.93110940595174
H 3.04029468975167 3.83000841924507 -3.21473446931026
H 3.29277125759252 2.70448558570725 -4.55470857149192
H 4.32747479976607 4.09874187288786 -1.13105297461626
H 5.62353159018936 2.96336050722238 -0.68603399028040
H 3.99800912914305 0.94742636170488 -2.99639591163043
H 5.06494102166534 2.34264381346925 -2.88646898883287
Ni 0.93016265099641 7.16713637439736 -1.31223927936165
C 0.51799063898786 8.97751976578490 -1.52892005183546
C 2.68117135058661 7.66792722418352 -0.83840787905132
C -0.82084600157112 6.66634553030577 -1.78606068735940
N 0.30042443867067 10.08904664257535 -1.66561164236216
N 3.73736929417124 7.99854233204121 -0.56112994611734
N -1.87696953453659 6.33565751857010 -2.06355179999022
H 4.27168972083079 3.27227082430478 0.43473808579292
H -2.54503094645933 -0.97570461055028 -1.34141326994329

1h, charge +2
Cu -0.00056328665989 0.00037733205406 0.00413448627972
Cu 2.38549693091784 1.85445407628747 -0.00473569545141
O 1.92522628606855 -0.00082929531030 0.00369894477081
O 0.45968734639372 1.85564075856187 -0.00429006435004
O -0.70228179708496 -0.23671124308331 2.21585199794627
O 3.08721543197314 2.09153269522963 -2.21644316677477
N -1.93702524585779 0.71644047270563 -0.21781762488157

N 4.32193888867778 1.13837103352349 0.21723649868848
N -0.28756860885028 -1.92168902447423 -0.22121432231349
N 2.67249224300322 3.77650044232958 0.22061319880373
C -0.36199403388490 -3.06005222829929 -0.23073485897188
C 2.74693768162614 4.91487372092915 0.23015369174650
C 2.99384204919720 -0.90883237967208 -0.06506330182443
C -0.60892840157336 2.76366383989469 0.06447210063015
H -0.53866761013022 0.55710548553763 2.74355514301765
C -2.11065218670290 -0.38472350597038 2.00653896596131
C -2.71313055371851 -0.08720267349749 -1.19750055047437
C -1.76540601893925 2.12910439774777 -0.68616623875923
C -2.62471036897269 0.71003960626975 1.10446787070814
C 4.15032965685183 -0.27429298286557 0.68556511084679
H 2.73394667490054 -1.86095000213377 0.40877712005831
H 3.25903058495713 -1.12162081340523 -1.10748492331926
H -0.34902727579465 3.71577161831817 -0.40938263493634
H -0.87411187715811 2.97645762105097 1.10689210240450
H -2.25090343007394 -1.37243657611681 1.57013866761237
H -2.64645964510374 -0.35648665805806 2.95796050885890
H -2.88377043814193 -1.08418963620336 -0.79788226592447
H -3.67834485948659 0.38820811241191 -1.39433079210641
H -1.52302262661032 2.08850922465575 -1.74834022321955
H -2.70225620732019 2.67999308905563 -0.55978472580130
H -2.45098223038255 1.66769955166953 1.59366961869651
H -3.70374727126886 0.60992666721586 0.95698600609937
H 2.92359702614680 1.29771698845864 -2.74414681178103
C 4.49558581263321 2.23953500020573 -2.00712009564525
C 5.09804420168250 1.94202413471598 1.19690938382325
C 5.00964400279259 1.14477180546352 -1.10506900194640
H 3.90797357395591 -0.23371622168039 1.74774668286966
H 5.08717249873075 -0.82518756693101 0.55915433138761
H 4.63583737350902 3.22724629589353 -1.57071568115487
H 5.03139470511291 2.21130871074219 -2.95854183417361
H 5.26867612709207 2.93901063421055 0.79728634223374
H 6.06326359883020 1.46662360562993 1.39373897651852
H 4.83591149557483 0.18711161206218 -1.59426728597742
H 6.08867755072247 1.24488279275747 -0.95757060730433
H -2.14960563454903 -0.16431024934743 -2.12568803012773
H 4.53452202338121 2.01913790284259 2.12509819385971
H -0.43753412261394 -4.13134191937595 -0.24556136541297
H 2.82249996614693 5.98616374799376 0.24460615881016

1i, charge +2
Cu 0.00000 0.00000 0.00000
Cu 2.38490 1.85559 -0.00002
O 1.92579 0.00000 0.00000
O 0.45909 1.85557 -0.00001
O -0.70207 -0.24743 2.21047
O 3.08697 2.10301 -2.21048
N -1.93686 0.71584 -0.21919
N 4.32174 1.13973 0.21919
N -0.28575 -1.92122 -0.23402
N 2.67064 3.77679 0.23400
C -0.35946 -3.05957 -0.24866
C 2.74437 4.91515 0.24866
C 2.99499 -0.90702 -0.07258
C -0.61011 2.76261 0.07258
H -0.54218 0.54046 2.74803
C -2.11030 -0.39539 2.00018
C -2.71224 -0.08389 -1.20264
C -1.76602 2.13069 -0.68116
C -2.62484 0.70308 1.10290
C 4.15091 -0.27512 0.68114
H 2.73544 -1.86199 0.39602
H 3.26176 -1.11609 -1.11565
H -0.35054 3.71753 -0.39612
H -0.87684 2.97173 1.11564
H -2.24800 -1.38009 1.55599
H -2.64657 -0.37300 2.95148
H -2.87455 -1.08499 -0.81004
H -3.68010 0.38848 -1.39404
H -1.52263 2.09504 -1.74338
H -2.70304 2.68152 -0.55424
H -2.45009 1.65849 1.59630
H -3.70403 0.60369 0.95543
H 2.92713 1.31530 -2.74832
C 4.49520 2.25096 -2.00017

C 5.09712 1.93947 1.20263
C 5.00974 1.15249 -1.10292
H 3.90760 -0.23951 1.74338
H 5.08791 -0.82595 0.55411
H 4.63295 3.23569 -1.55607
H 5.03146 2.22853 -2.95148
H 5.25931 2.94060 0.81003
H 6.06505 1.46715 1.39381
H 4.83499 0.19711 -1.59638
H 6.08893 1.25186 -0.95548
H -2.15052 -0.15206 -2.13264
H 4.53527 2.00765 2.13254
C -0.46683 -4.49973 -0.27277
H 0.49224 -4.94068 0.00213
H -0.74502 -4.82575 -1.27595
H -1.23117 -4.81587 0.43861
C 2.85212 6.35478 0.27436
H 3.59193 6.67451 -0.46096
H 3.16322 6.67800 1.26872
H 1.88425 6.79639 0.03321

2a, charge -6
Cu 0.00000 0.00000 0.00000
Cu 2.33795 1.88922 0.02489
O 0.39462 1.88922 0.02489
O 1.94333 -0.00000 0.00000
N -1.83370 0.60715 -0.68304
N -0.18019 -1.89896 -0.52816
N -0.30604 -0.25199 2.19327
C -0.54408 2.68806 -0.62059
C 2.88203 -0.79884 0.64547
C -2.96712 -0.09669 -0.05123
C -1.88465 2.06223 -0.36320
C -1.88771 0.39700 -2.14672
C -0.12547 -2.89093 -1.09391
C 0.06474 -0.46830 3.24944
N 4.17165 1.28207 0.70793
N 2.51814 3.78818 0.55304
N 2.64399 2.14120 -2.16838
H -0.38921 2.78448 -1.49113
H -0.52827 3.62885 -0.36782
C 4.22259 -0.17302 0.38809
H 2.72716 -0.89527 1.51601
H 2.86621 -1.73963 0.39270
H -2.95438 -1.02117 -0.30860
H -3.79143 0.30321 -0.33648
H -2.89336 -0.02940 0.90455
H -2.07658 2.10570 0.56503
H -2.57704 2.45494 -0.85069
H -1.14373 0.84040 -2.56102
H -2.70759 0.75699 -2.49094
H -1.84700 -0.54255 -2.33608
Ni -0.02202 -4.43751 -2.12939
Ni 0.65166 -0.91223 4.96608
C 5.30506 1.98590 0.07611
C 4.22566 1.49222 2.17160
C 2.46341 4.78014 1.11879
C 2.27320 2.35751 -3.22456
H 4.41453 -0.21649 -0.54015
H 4.91499 -0.56572 0.87558
C 0.08143 -5.98408 -3.16486
C 0.42960 -5.49284 -0.65606
C -0.47364 -3.38217 -3.60272
C 1.46470 0.74785 5.20670
C -0.16138 -2.57232 4.72546
C 1.23857 -1.35617 6.68273
H 5.29233 2.91038 0.33348
H 6.12937 1.58601 0.36136
H 5.23131 1.91862 -0.87967
H 3.48168 1.04881 2.58591
H 5.04553 1.13222 2.51583
H 4.18496 2.43176 2.36097
Ni 2.35996 6.32672 2.15427
Ni 1.68629 2.80145 -4.94119
N 0.13616 -6.97604 -3.73062
N 0.69553 -6.18300 0.21155
N -0.73957 -2.69201 -4.47032

N 1.96114 1.75201 5.43532
N -0.65782 -3.57648 4.49684
N 1.60936 -1.57248 7.73889
C 2.25652 7.87330 3.18975
C 2.81158 5.27138 3.62760
C 1.90835 7.38206 0.68093
N 2.20179 8.86526 3.75550
N 3.07751 4.58122 4.49521
N 1.64241 8.07222 -0.18666
C 2.49933 4.46153 -4.70057
C 0.87325 1.14136 -5.18182
C 1.09937 3.24539 -6.65784
N 2.99577 5.46570 -4.47196
N 0.72859 3.46170 -7.71401
N 0.37681 0.13720 -5.41044
H 2.00322 -0.63083 -2.13250
H 0.33473 2.52004 2.15739
O -0.02948 2.90704 2.82109
H 0.56224 3.09256 3.40074
O 2.36743 -1.01782 -2.79621
H 1.77571 -1.20334 -3.37585

2b, charge -6
Cu 0.00000 0.00000 0.00000
Cu 1.88699 2.32746 -0.24065
O 0.00000 1.93015 0.00000
O 1.88699 0.39731 -0.24065
N -1.98926 0.21056 -0.44289
N 0.14789 -1.90234 -0.52604
N 0.02592 -0.28093 2.21088
C -1.15475 2.51181 -0.51399
C 3.04174 -0.18435 0.27335
C -2.86768 -0.70192 0.31535
C -2.29662 1.62850 -0.10061
C -2.17997 -0.02504 -1.89118
C 0.33379 -2.86937 -1.10685
C 0.56096 -0.40323 3.21016
N 3.87625 2.11690 0.20224
N 1.73910 4.22981 0.28538
N 1.86107 2.60840 -2.45153
H -1.10129 2.61273 -1.60583
H -1.30616 3.51974 -0.10905
C 4.18362 0.69897 -0.14003
H 2.98828 -0.28527 1.36518
H 3.19315 -1.19228 -0.13160
H -2.61966 -1.73097 0.06288
H -3.91749 -0.51475 0.06700
H -2.71513 -0.54711 1.38138
H -2.41376 1.67726 0.98212
H -3.24455 1.92779 -0.56144
H -1.52077 0.61950 -2.46816
H -3.21786 0.17954 -2.17280
H -1.94199 -1.06051 -2.12063
Ni 0.62223 -4.37535 -2.16687
Ni 1.43419 -0.69562 4.83508
C 4.75468 3.02939 -0.55601
C 4.06696 2.35251 1.65053
C 1.55320 5.19684 0.86620
C 1.32603 2.73070 -3.45080
H 4.30076 0.65020 -1.22277
H 5.13154 0.39968 0.32079
C 0.91068 -5.88133 -3.22689
C 1.45896 -5.29698 -0.77443
C -0.21449 -3.45373 -3.55932
C 1.91404 1.09858 4.99439
C 0.95434 -2.48983 4.67577
C 2.30741 -0.98802 6.46002
H 4.50664 4.05843 -0.30356
H 5.80449 2.84224 -0.30763
H 4.60214 2.87455 -1.62203
H 3.40776 1.70796 2.22751
H 5.10486 2.14792 1.93215
H 3.82898 3.38798 1.87999
Ni 1.26476 6.70282 1.92623
Ni 0.45281 3.02309 -5.07573
N 1.09658 -6.84836 -3.80772
N 1.96572 -5.90694 0.04466

N -0.72125 -2.84376 -4.37841
N 2.21909 2.18589 5.17261
N 0.64929 -3.57714 4.49756
N 2.84245 -1.11031 7.45928
C 0.97632 8.20879 2.98625
C 2.10149 5.78119 3.31867
C 0.42803 7.62444 0.53378
N 0.79042 9.17583 3.56706
N 2.60825 5.17123 4.13776
N -0.07873 8.23441 -0.28531
C 0.93266 4.81729 -4.91642
C -0.02704 1.22888 -5.23504
C -0.42042 3.31548 -6.70066
N 1.23771 5.90461 -4.73821
N -0.95546 3.43777 -7.69993
N -0.33210 0.14157 -5.41325
H 1.99156 -0.14926 -2.20728
H -0.10457 2.47671 1.96663
O -0.27368 2.87574 2.83999
H 0.44144 2.54754 3.40210
O 2.16067 -0.54827 -3.08065
H 1.44555 -0.22007 -3.64275

2c, charge +6
Cu 0.00000 0.00000 0.00000
Cu 2.33794 1.88921 0.02497
O 0.39461 1.88921 0.02497
O 1.94333 -0.00000 0.00000
N -1.83370 0.60718 -0.68302
N -0.18020 -1.89894 -0.52825
N -0.30605 -0.25208 2.19325
N 2.64399 2.14129 -2.16828
N 2.51814 3.78815 0.55320
N 4.17164 1.28203 0.70799
C 2.27320 2.35765 -3.22445
C -0.12548 -2.89089 -1.09404
C 0.06474 -0.46844 3.24942
C 2.46341 4.78010 1.11901
Ni -0.02206 -4.43746 -2.12954
Ni 0.65164 -0.91244 4.96603
C -0.54409 2.68809 -0.62048
C 2.88203 -0.79888 0.64545
C -2.96713 -0.09668 -0.05123
C -1.88465 2.06225 -0.36311
C -1.88772 0.39709 -2.14670
H -0.33633 2.76003 -1.69568
H -0.53305 3.70984 -0.22331
C 4.22259 -0.17304 0.38809
H 2.67426 -0.87086 1.72064
H 2.87099 -1.82062 0.24824
H -2.90702 -1.15791 -0.28470
H -3.91646 0.29917 -0.42605
H -2.92047 0.03778 1.02751
H -2.12410 2.14764 0.69708
H -2.68582 2.54222 -0.93538
H -1.03502 0.87203 -2.62617
H -2.81100 0.82131 -2.55319
H -1.86038 -0.66849 -2.35856
C 5.30507 1.98589 0.07619
C 4.22566 1.49212 2.17167
H 4.46204 -0.25844 -0.67212
H 5.02377 -0.65302 0.96033
C 0.08151 -5.98408 -3.16496
C 0.42955 -5.49248 -0.65598
C -0.47365 -3.38203 -3.60285
C 1.46469 0.74763 5.20675
C -0.16141 -2.57249 4.72527
C 1.23848 -1.35658 6.68266
H 5.24496 3.04712 0.30966
H 6.25440 1.59003 0.45100
H 5.25839 1.85144 -1.00255
H 3.37299 1.01713 2.65114
H 5.14896 1.06795 2.57815
H 4.19825 2.55769 2.38354
Ni 2.36000 6.32666 2.15452
Ni 1.68629 2.80165 -4.94106
N 0.13633 -6.97605 -3.73069

N 0.69550 -6.18233 0.21187
N -0.73958 -2.69182 -4.47041
N 1.96113 1.75178 5.43540
N -0.65787 -3.57663 4.49656
N 1.60921 -1.57307 7.73881
C 2.25654 7.87330 3.18990
C 2.81159 5.27123 3.62782
C 1.90829 7.38173 0.68103
N 2.20180 8.86531 3.75557
N 3.07751 4.58102 4.49539
N 1.64227 8.07162 -0.18675
C 2.49934 4.46171 -4.70033
C 0.87325 1.14158 -5.18177
C 1.09946 3.24573 -6.65771
N 2.99579 5.46586 -4.47165
N 0.72873 3.46216 -7.71386
N 0.37681 0.13743 -5.41043
H 2.21728 -0.58230 -1.93655
H 0.12069 2.47198 1.96128
O -0.02949 2.90692 2.82121
H 0.84204 2.92649 3.22937
O 2.36743 -1.01771 -2.79625
H 1.49558 -1.03959 -3.20358
H -1.09657 -4.46678 4.31708
H 1.93937 -1.78300 8.66861
H 2.26838 2.56016 5.94481
H 3.58107 4.17116 5.26647
H 2.14938 9.72995 4.27304
H 1.39640 8.66908 -0.96161
H 3.43447 6.35602 -4.29215
H 0.39853 3.67192 -8.64368
H 0.07004 -0.67116 -5.91979
H -1.24310 -2.28172 -5.24139
H 0.18885 -7.84068 -4.24816
H 0.94118 -6.77960 0.98693

Cu–NC–Ni–CN–Cu fragment, charge +2
Ni 5.33882467663953 -5.21940787259212 -0.01381448983637
C 5.08547716155685 -5.60954039741194 1.79669035351638
C 5.59217219148046 -4.82927534809138 -1.82431933329171
C 6.84914884643188 -6.33947549125855 -0.08925045139519
C 3.82850050684719 -4.09934025392568 0.06162147172245
N 4.91286564588052 -5.81790500304185 2.90497953575484
N 5.76478370842096 -4.62091074083548 -2.93260851502758
N 7.76396090450371 -7.01715902275257 -0.16423207256709
N 2.91368845230219 -3.42165671783128 0.13660309434509
Cu 6.30830026778615 -4.34736084875980 -4.79196185682405
O 7.01089392080766 -6.10605759256583 -5.14129127564972
O 6.69124534499776 -4.32513499033813 -6.66460351549273
O 8.06547501989073 -3.03394939848595 -3.99974499242951
N 5.48230167340060 -2.48073721830911 -5.17321280942901
C 6.47353984230074 -3.10889868103212 -7.33133628360203
H 8.74648389385380 -2.80613724494776 -4.64787294900338
C 7.34024973423140 -1.85266484509973 -3.64219037655775
C 4.19804676538453 -2.28818769618243 -4.45102375851709
C 5.28214505930327 -2.45291930780051 -6.65765798977387
C 6.45118517737596 -1.41819363120955 -4.78116340301495
H 6.24993410075974 -3.29177131126013 -8.38381301067380
H 7.36762051698067 -2.48578056830364 -7.27019080772293
H 6.76403297486995 -2.10745513617706 -2.75439250891257
H 8.02700068461746 -1.04357868025481 -3.38622672605446
H 4.38889219753543 -2.24860742505405 -3.38175818478374
H 3.73029317174038 -1.35407927186020 -4.77242381458837
H 4.37862249603408 -3.02289949599771 -6.87519726299050
H 5.14605399788712 -1.42784806293575 -7.00797291973339
H 7.08100953707141 -1.18324981782282 -5.63814223121278
H 5.90397246857964 -0.50956508416276 -4.51672377699725
Cu 4.36934908795779 -6.09145489465573 4.76433287804090
O 3.98640399993937 -6.11368072832872 6.63697454027404
O 3.66675544938793 -4.33275814364016 5.11366228963584
O 2.61217432444581 -7.40486632706259 3.97211599316655
N 5.19534762001449 -7.95807855983633 5.14558382500729
C 4.20410948492669 -7.32991699358223 7.30370739637220
H 1.93076196403700 -7.63278337382730 4.61977830049906
C 3.33739961536573 -8.58615087539495 3.61456137646386
C 6.47960255468323 -8.15062818583398 4.42339484918535
C 5.39550417604157 -7.98589643870418 6.63002900805322

```
C 4.22646410325256 -9.02062213291207 4.75353443222927
H 4.42775850520111 -7.14702503809426 8.35617295263992
H 3.31000972718705 -7.95300459625886 7.24259407769412
H 6.28877090988986 -8.19015047772940 3.35412438014848
H 6.94732564401452 -9.08477098121384 4.74474109039787
H 3.59663137381732 -9.25554177164684 5.61051321097052
H 4.77368019284748 -9.92925355596345 4.48911422325872
H 6.29900602438180 -7.41588161588691 6.84756991878066
H 5.53163956142398 -9.01096293475725 6.98033791555789
H 3.91362491754675 -8.33137016330409 2.72676428056283
H 2.65065197632607 -9.39523345411994 3.35858662533946
H 4.29585679087545 -3.75196829779385 5.57106248273675
H 3.33170976320688 -3.81576301345136 4.36410667175155
H 3.15856134207704 -5.68842947312446 6.90683562710512
H 7.34684634624299 -6.62271323637738 -4.39190776820392
H 6.38133763823104 -6.68710855646838 -5.59773621637836
H 7.51882322841380 -4.75074095403810 -6.93469549373476
H 7.14214768298894 -7.31479909859716 4.63932282304324
H 3.53552505580318 -3.12404897109476 -4.66690082985467
```

References.

S1. Karaağaç, D. VIBRATIONAL SPECTROSCOPIC AND THERMAL INVESTIGATIONS OF THE CYANO-BRIDGED POLYMERIC COMPLEXES WITH 3-AMINOMETHYLPYRIDINE. *Eskişehir Techn. Univ. J. Sci. Technol. A.* **2019**, *20*, 216–226.

S2. Rodríguez-Hernández, J.; Lemus-Santana, A. A.; Vargas, C. N.; Reguera, E. Three Structural Modifications in the Series of Layered Solids T(H2O)2[Ni(CN)4]·xH2O with T = Mn, Co, Ni: Their Nature and Crystal Structures. *Compt. Rend. Chim.* **2012**, *15*, 350–355.

S3. Qin, Y. L.; Yang, B. W.; Wang, G. F.; Sun, H. A Cyanide-Bridged Heterometallic Coordination Polymer Constructed from Square-Planar [Ni(CN)4]2-: Synthesis, Crystal Structure, Thermal Decomposition, Electron Paramagnetic Resonance (EPR) Spectrum and Magnetic Properties. *Acta Crystallogr. C.* **2016**, *72*, 555–560.

S4. Nakamoto, K. Infrared and Raman Spectra of Inorganic and Coordination Compounds, 4th ed.; Wiley-Interscience: New York, 1978.

S5. Gruenwald, K. R.; Kirillov, A. M.; Haukka, M.; Sanchiz, J.; Pombeiro, A. J. L. Mono-, Di- and Polynuclear Copper(II) Compounds Derived from N-Butyldiethanolamine: Structural Features, Magnetism and Catalytic Activity for the Mild Peroxidative Oxidation of Cyclohexane. *Dalton Trans.* **2009**, *12*, 2109–2120.
